# Identification and molecular characterization of cellular factors required for glucocorticoid receptor-mediated mRNA decay

**DOI:** 10.1101/gad.286484.116

**Published:** 2016-09-15

**Authors:** Ok Hyun Park, Joori Park, Mira Yu, Hyoung-Tae An, Jesang Ko, Yoon Ki Kim

**Affiliations:** 1 Creative Research Initiatives Center for Molecular Biology of Translation, Korea University, Seoul 02841, Republic of Korea;; 2 Division of Life Sciences, Korea University, Seoul 02841, Republic of Korea

**Keywords:** glucocorticoid receptor-mediated mRNA decay, YBX1, HRSP12, UPF1, PNRC2

## Abstract

Glucocorticoid (GC) receptor (GR) has been shown recently to bind a subset of mRNAs and elicit rapid mRNA degradation. However, the molecular details of GR-mediated mRNA decay (GMD) remain unclear. Here, we demonstrate that GMD triggers rapid degradation of target mRNAs in a translation-independent and exon junction complex-independent manner, confirming that GMD is mechanistically distinct from nonsense-mediated mRNA decay (NMD). Efficient GMD requires PNRC2 (proline-rich nuclear receptor coregulatory protein 2) binding, helicase ability, and ATM-mediated phosphorylation of UPF1 (upstream frameshift 1). We also identify two GMD-specific factors: an RNA-binding protein, YBX1 (Y-box-binding protein 1), and an endoribonuclease, HRSP12 (heat-responsive protein 12). In particular, using HRSP12 variants, which are known to disrupt trimerization of HRSP12, we show that HRSP12 plays an essential role in the formation of a functionally active GMD complex. Moreover, we determine the hierarchical recruitment of GMD factors to target mRNAs. Finally, our genome-wide analysis shows that GMD targets a variety of transcripts, implicating roles in a wide range of cellular processes, including immune responses.

## Abstract (precis)

In this study, Park et al. investigated the molecular mechanisms regulating glucocorticoid receptor-mediated mRNA decay (GMD). The authors characterize the molecular details of GMD, identify specific factors required for efficient GMD, and perform RNA sequencing, identifying many endogenous GMD substrates.

Glucocorticoid (GC) functions in a variety of physiological and developmental events, including glucose metabolism, anti-inflammation, fetal development, and brain function (Oakley and Cidlowski 2011; Santos et al. 2011; Vandevyver et al. 2012). At the molecular level, the functions of GC are mediated by binding to the GC receptor (GR), a typical nuclear receptor (Kato et al. 2011; Lonard and O'Malley 2012). A ligand-unbound form of GR is located largely in the cytoplasm as a component of a complex containing chaperones, cochaperones, and immunophilins. When GR binds to GC, however, it dissociates from the complex, and the ligand-bound GR then enters the nucleus. In the nucleus, ligand-bound GR binds to a specific *cis*-acting GC response element and *trans*-acting coregulatory proteins, leading to transcriptional activation or repression.

Although it has long been appreciated that GR functions as a DNA-binding transcription factor, several recent reports demonstrated the ability of GR to bind a subset of RNAs (Dhawan et al. 2007; Kino et al. 2010; Ishmael et al. 2011; Dhawan et al. 2012; Cho et al. 2015). Unlike GR binding to DNAs, GR associates with mRNAs even in the absence of a ligand. Upon binding to a ligand, the GR, which is preloaded on mRNAs, recruits proline-rich nuclear receptor coregulatory protein 2 (PNRC2). PNRC2 provides a binding platform for upstream frameshift 1 (UPF1) and decapping enzyme 1a (DCP1A), the latter of which is a component of the decapping complex. Eventually, the resulting complex triggers rapid degradation of target mRNAs, termed “GR-mediated mRNA decay” (GMD) (Cho et al. 2015).

It is known that GMD factors PNRC2 and UPF1 are commonly involved in multiple types of mRNA decay pathway (Cho et al. 2009, 2012, 2013; Lai et al. 2012; Choe et al. 2014a): nonsense-mediated mRNA decay (NMD), which specifically recognizes and removes aberrant mRNAs containing a premature termination codon (PTC); staufen-mediated mRNA decay (SMD), which triggers rapid degradation of mRNAs containing the staufen-binding site in the 3′ untranslated region (UTR); and replication-dependent histone mRNA decay (HMD), which is an mRNA degradation pathway activated during the cell cycle (Marzluff et al. 2008; Karam et al. 2013; Fatscher et al. 2015; Lykke-Andersen and Jensen 2015; Karousis et al. 2016). It should be noted that NMD, SMD, and HMD are dependent on a translation event.

Although all of these pathways share PNRC2 and UPF1 as common factors, GMD is mechanistically different from NMD in several respects (Cho et al. 2015). First, NMD is tightly coupled to translation because an elongating ribosome has to recognize a PTC on the mRNA; in contrast, our previous data using a GMD reporter mRNA harboring a strong stem–loop structure suggest that GMD could occur independently of translation. Second, in the case of NMD, UPF1 is recruited to a ribosome terminating at a PTC. The UPF1-mediated selective recognition of target substrates is reinforced by the interaction of UPF1 and a PTC-distal exon junction complex (EJC), which is deposited onto mRNA as a consequence of splicing in the nucleus. UPF1 is then hyperphosphorylated by the NMD-specific kinase SMG1, one of the phosphatidylinositol 3-kinase-related kinases (PI3KK). The hyperphosphorylated UPF1 recruits PNRC2 and SMG5–7, eliciting rapid mRNA degradation. Unlike NMD, on the other hand, target recognition and mRNA degradation are separable in GMD. GR is preloaded onto target substrates; however, this complex remains inactive for mRNA degradation. Only once the preloaded GR binds to GC does the GR recruit PNRC2 and UPF1, consequently eliciting rapid mRNA degradation. Third, with respect to ligand dependency, GMD is an inducible type of RNA decay, whereas NMD is constitutively active for monitoring mRNA quality. Fourth, NMD and GMD require their own specific factors for efficient mRNA degradation. For instance, a conventional NMD, unlike GMD, requires UPF2.

Here, we characterize the molecular details of GMD. Our data show that, unlike NMD, GMD occurs independently of a translation event, EJC, and previously well-characterized UPF1-interacting NMD-specific factors such as SMG1 kinase and SMG5–7. Instead, efficient GMD requires helicase activity, ATM-mediated hyperphosphorylation, and N-terminal 1–72 amino acids of UPF1, a critical region for PNRC2 binding. In addition, we identify two specific GMD factors: an RNA-binding protein, Y-box-binding protein 1 (YBX1), and an endoribonuclease, heat-responsive protein 12 (HRSP12; also known as UK114 antigen homolog and 14.5-kDa translational inhibitor protein). Using immunoprecipitation, complementation experiments, and artificial tethering of GMD factors to the 5′ UTR of reporter mRNAs, we determined the functional hierarchy of GMD factors. In addition, we provide genome-wide evidence indicating that GMD plays a role in a wide range of physiological pathways, including immune cell responses.

## Results

### GMD occurs independently of a translation event and EJC

To clearly compare GMD and NMD with respect to translation dependency, we employed two approaches in this study: the iron-responsive element (IRE)/iron regulatory protein (IRP) system and specific down-regulation of translation termination factors using specific siRNAs. The IRE/IRP system is a well-defined system for the control of translation, the efficiency of which depends on intracellular iron concentration. In the absence of intracellular iron, cytosolic IRP binds to IRE in the 5′ UTR and blocks ribosome recruitment. In the presence of sufficient intracellular iron, on the other hand, IRP dissociates from IRE, and, consequently, ribosomes can easily access the 5′ end of mRNA to initiate translation.

**Figure 1.**
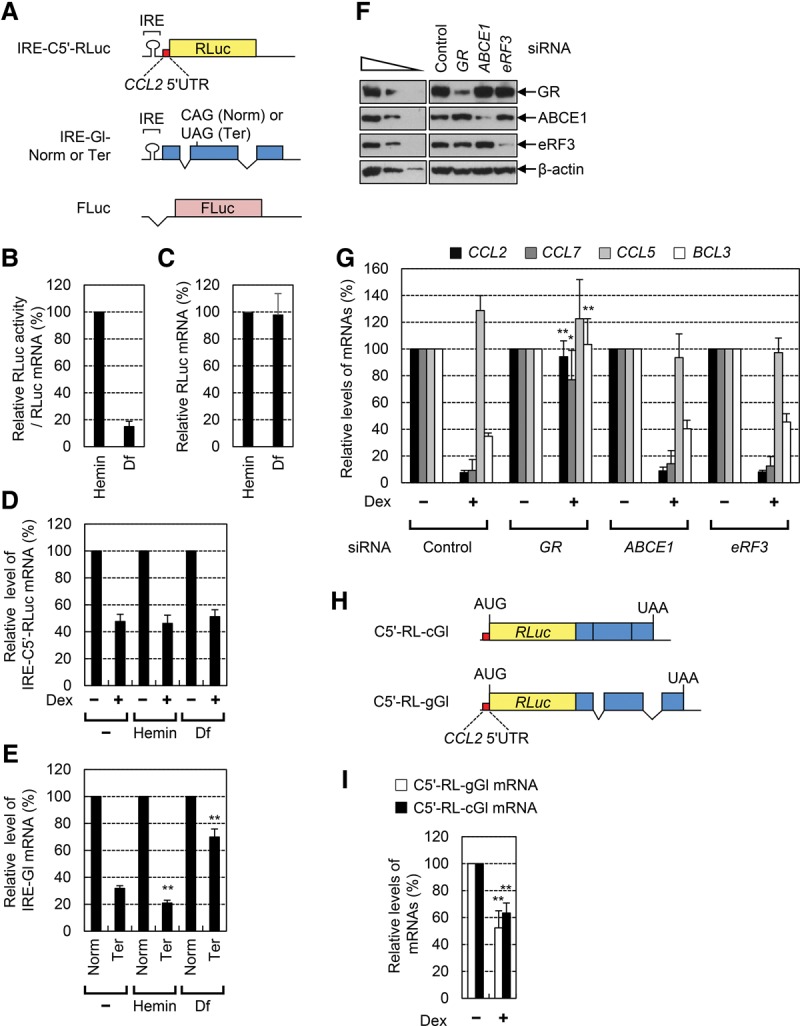
GMD is a unique mRNA decay pathway, which occurs independently of a translation event and EJC. (*A*) A schematic of IRE-containing GMD and NMD reporter constructs. Details are described in the Results. (*B*–*E*) The effects of a translation event on GMD and NMD. HeLa cells transiently expressing either GMD or NMD reporter mRNAs and FLuc mRNA were either treated or not treated with hemin or desferal (Df) for 18 h before harvesting. (*B*,*C*) The change in translational efficiency (*B*) and the level (*C*) of reporter mRNA depending on intracellular iron concentration. The RLuc activities were normalized to FLuc activities. The levels of RLuc mRNAs were also normalized to the levels of FLuc mRNAs. Translational efficiencies were calculated by dividing the normalized RLuc activities by normalized RLuc mRNAs. (*D*) Relative levels of GMD reporter mRNAs. The cells were either treated or not treated with dexamethasone (Dex) 12 h before harvesting. The levels of IRE-C5′-RLuc mRNAs were normalized to the levels of FLuc mRNAs. The normalized levels of IRE-C5′-RLuc mRNAs in the cells untreated with Dex were arbitrarily set to 100%. (*E*) Relative levels of NMD reporter mRNAs. The levels of IRE-Gl mRNAs either Norm or Ter were normalized to the levels of FLuc mRNAs. The normalized levels of IRE-Gl-Norm mRNAs were arbitrarily set to 100%. (*F*,*G*) The effects of down-regulation of ATP-binding cassette subfamily E member 1 (ABCE1) and eukaryotic translation release factor 3 (eRF3) on GMD. (*F*) Specific down-regulation of GR, ABCE1, and eRF3 was demonstrated by Western blotting. For the purpose of quantitative comparison, threefold serial dilutions of total cell extracts were loaded in the three *left* lanes. (*G*) The levels of endogenous GMD substrates and a control mRNA (*CCL5* mRNA) were normalized to endogenous *GAPDH* mRNAs. (*H*) A schematic of GMD reporter constructs either containing or not containing introns. (*I*) The effect of an intron and EJC on GMD. The columns and bars in each panel represent the mean and standard deviation of three independent biological replicates. *n* = 3. Two-tailed, equal-sample variance Student's *t*-tests were used to calculate the *P*-values. (**) *P* < 0.01; (*) *P* < 0.05.

For the IRE/IRP system, we generated a GMD reporter construct in which an IRE was inserted immediately upstream of the *CCL2* 5′ UTR (C5′) followed by the ORF of Renilla luciferase (RLuc) cDNA (Fig. 1A). The *CCL2* 5′ UTR is known to be sufficient for eliciting efficient GMD in the presence of a ligand because this region contains a GR-binding site. As a control, we used another construct encoding the ORF of firefly luciferase (FLuc) cDNA lacking both an IRE and the *CCL2* 5′ UTR. For comparison, we also generated NMD reporter constructs in which an IRE was inserted into the 5′ UTR of β-globin (Gl) followed by Gl genomic sequences containing either a normal codon (Norm) or PTC (Ter) at the position of the 39th amino acid.

HeLa cells were transfected with either the GMD or the NMD reporter construct, and the relative level of each reporter mRNA was measured using quantitative real-time RT–PCR (qRT–PCR). Our IRE/IRP system worked correctly, as demonstrated by a drastic decrease in RLuc activity from the cells expressing GMD reporter mRNA upon desferal (Df; an iron chelator) treatment compared with hemin (an iron resource) treatment (Fig. 1B) without a change in mRNA levels (Fig. 1C). Under the same conditions, the cells were treated with dexamethasone (Dex; a chemical derivative of GC) to induce GMD. The results showed that Dex treatment caused an approximately twofold decrease in the levels of GMD reporter mRNAs, indicating efficient GMD (Fig. 1D). Intriguingly, the efficiency of GMD was not significantly affected by hemin or Df treatment. In contrast, the levels of IRE-containing NMD reporter mRNAs were decreased by 1.5-fold and increased by twofold upon treatment with hemin and Df, respectively (Fig. 1E).

As another approach, HeLa cells were depleted of either endogenous eukaryotic translation release factor 3 (eRF3) or ATP-binding cassette subfamily E member 1 (ABCE1), which interact with each other and play an important role in translation termination and ribosome recycling in eukaryotic cells (Khoshnevis et al. 2010; Pisarev et al. 2010; Dever and Green 2012). Levels of previously identified endogenous GMD substrates (*CCL2* mRNA, *CCL7* mRNA, and *BCL3* mRNA) were then measured by qRT–PCR. Specific down-regulation was confirmed by Western blotting (Fig. 1F). qRT–PCR results showed that, although down-regulation of GR almost completely abolished GMD, down-regulation of either ABCE1 or eRF3 did not significantly affect GMDs of known substrates (Fig. 1G). The level of *CCL5* mRNA, which lacks a GR-binding site and served as a negative control, was not affected by Dex treatment or down-regulation of GR, ABCE1, or eRF3. Of note, down-regulation of ABCE1 inhibited NMDs of Gl and GPx1 mRNAs to the same extent as down-regulation of UPF1 (Supplemental Fig. S1). All of these data indicate that, unlike NMD, GMD does not require translation termination factors and occurs independently of a translation event. In support of these conclusions, our previous data showed that artificial insertion of a strong stem–loop structure into the 5′ UTR of GMD reporter mRNA does not affect GMD efficiency, although translational efficiency of the reporter mRNA is drastically reduced (Cho et al. 2015).

To further elucidate the molecular properties of GMD, we investigated a possible effect of EJC on GMD. To this end, we generated two GMD reporter constructs, C5′-RL-cGl and C5′-RL-gGl, which contained, in sequential order, *CCL2* 5′ UTR, the ORF of RLuc lacking a translation termination codon, and either cDNA (c) or genomic (g) sequence of the Gl gene (Fig. 1H). Although both GMD reporters generate identical mRNAs in their sequences, C5′-RL-gGl will have two EJCs as a result of pre-mRNA splicing. The results showed that Dex treatment reduced the levels of reporter mRNAs comparably (Fig. 1I), indicating that GMD occurs independently of EJC and pre-mRNA splicing. Since it is well known that EJC enhances mRNA translation (Nott et al. 2004), these data also support the idea that GMD occurs independently of a translation event.

### ATPase/helicase activity and ATM-mediated hyperphosphorylation of UPF1 are critical for efficient GMD

The ATPase/helicase activity of UPF1 is known to be critical for disassembly of NMD factors from mRNAs undergoing NMD (Franks et al. 2010). In addition, a continuous cycle of UPF1 phosphorylation by SMG1 kinase and UPF1 dephosphorylation by phosphatase 2A is essential for efficient NMD (Karam et al. 2013; Fatscher et al. 2015; Lykke-Andersen and Jensen 2015). In particular, a phosphorylated form of UPF1 more strongly associates with adaptors or effectors such as PNRC2 and SMG5–7 and triggers rapid mRNA degradation in NMD (Cho et al. 2009, 2013).

**Figure 2.**
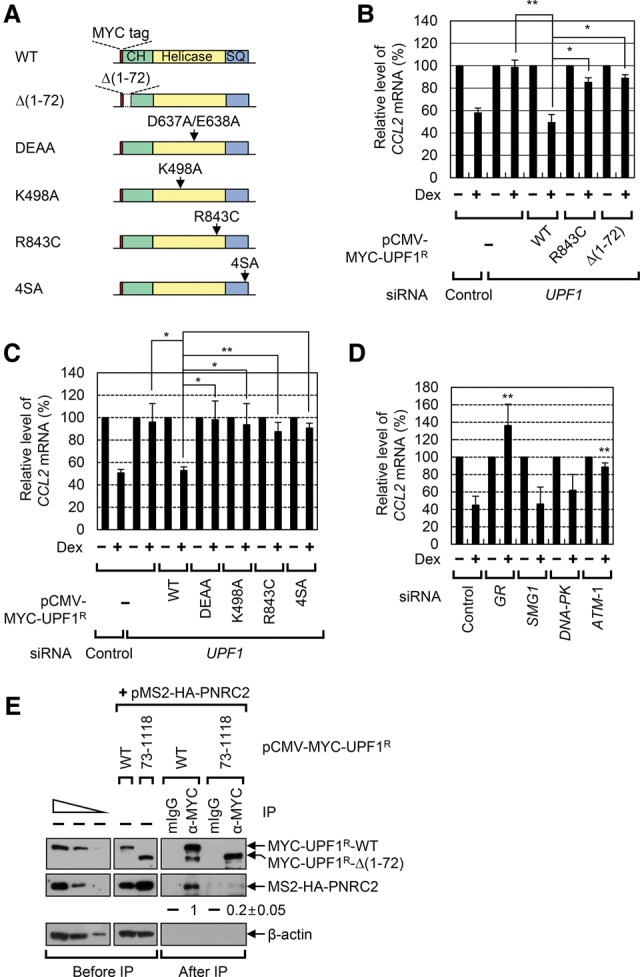
GMD requires ATPase/helicase activity, hyperphosphorylation, and the binding ability to PNRC2 of UPF1. (*A*) A schematic diagram of MYC-tagged UPF1^R^ wild type and its variants. (*B*,*C*) Complementation experiments using *UPF1* siRNA and MYC-UPF1^R^ wild type and its variants. The levels of endogenous *CCL2* mRNA, which were normalized to the levels of endogenous *GAPDH* mRNA, in the cells not treated with Dex for 1 h were arbitrarily set to 100%. *n* = 2 for *B*; *n* = 3 for *C*. (**) *P* < 0.01; (*) *P* < 0.05. (*D*) The effect of ATM down-regulation on GMD. HeLa cells were depleted of the indicated protein using siRNA, and the levels of endogenous *CCL2* mRNA were measured using qRT–PCR. *n* = 3. (*E*) Immunoprecipitations of MYC-UPF1^R^ wild type and MYC-UPF1^R^Δ(1–72). HEK293T cells were transiently cotransfected with MS2-HA-PNRC2 and either MYC-UPF1^R^ wild type or MYC-UPF1^R^Δ(1–72). Immunoprecipitations were performed using either α-MYC antibody or a nonspecific mouse IgG (mIgG). The samples were analyzed by Western blotting before and after immunoprecipitation. The levels of coimmunoprecipitated MS2-HA-PNRC2 were normalized to the level of immunoprecipitated MYC-UPF1^R^. The normalized level obtained from the immunoprecipitation of MYC-UPF1^R^ wild type was arbitrarily set to 1.0. *n* = 2.

To investigate which molecular features of UPF1 are involved in GMD, we performed complementation experiments in which cells were depleted of UPF1 using *UPF1* siRNA and exogenously expressed siRNA-resistant (R) UPF1 wild type or variants. The UPF1^R^ variants used in this study were (1) Δ(1–72), which lacks N-terminal 1–72 amino acids; (2) DEAA, which cannot hydrolyze ATP; (3) K498A, which fails to bind ATP; (4) R843C, which lacks helicase activity; and (5) 4SA, which contains amino acid substitutions from serine to alanine at preferentially phosphorylated residues 1073, 1078, 1096, and 1116 (Fig. 2A). Specific down-regulation of UPF1 by siRNA and comparable expression of UPF1^R^ wild type and variants were confirmed by Western blotting (Supplemental Fig. S2A,B). Complementation results showed that the exogenous UPF1^R^ wild type completely restored GMD of endogenous *CCL2* mRNA (Fig. 2B,C). On the other hand, all variants tested failed to restore GMD of *CCL2* mRNA, indicating that N-terminal 1–72 amino acids, ATPase/helicase ability, and phosphorylation of UPF1 are important for efficient GMD.

Because our previous data showed that down-regulation of SMG1 does not affect GMD (Cho et al. 2015), it was quite unexpected that UPF1^R^-4SA failed to restore GMD in our complementation experiments (Fig. 2C). To explain this discrepancy, we investigated the effect of other PI3KKs on GMD. Down-regulation of ATM, but not of SMG1 or DNA-PKcs, significantly inhibited Dex-induced mRNA degradation of *CCL2* mRNA (Fig. 2D; Supplemental Fig. S2C–E). All data indicate that, whereas NMD preferentially uses SMG1, GMD preferentially uses ATM-mediated UPF1 phosphorylation.

### Efficient GMD requires an interaction between UPF1 and PNRC2 via 1–72 amino acids of UPF1

It is known that the N-terminal region of UPF1 interacts with several adaptors and effectors, including PNRC2, UPF2, and SMG6 (Mendell et al. 2000; Cho et al. 2009, 2012; Okada-Katsuhata et al. 2012). In addition, our previous report revealed that down-regulation of PNRC2, but not UPF2, inhibits GMD (Cho et al. 2015). Therefore, we tested whether SMG6 contributes to GMD via its interaction with 1–72 of UPF1. To this end, we investigated the effect of SMG5–7 down-regulation on GMD (Supplemental Fig. S2F,G). The results showed that, whereas GR down-regulation almost completely blocked GMD, down-regulation of SMG6 or SMG6-related proteins (SMG5 and SMG7) had no significant effect on the GMD of endogenous substrates. In addition, our immunoprecipitation results showed that MS2-HA-PNRC2 was preferentially enriched in immunoprecipitation of MYC-UPF1 wild type compared with immunoprecipitation of MYC-UPF1-Δ(1–72) (Fig. 2E). Considering that down-regulation of PNRC2 inhibits GMD (Cho et al. 2015), all data indicate that the interaction between 1–72 of UPF1 and PNRC2, rather than UPF2 or SMG6, is critical for efficient GMD.

### Identification of YBX1 and HRSP12 as functional components of GMD

**Figure 3.**
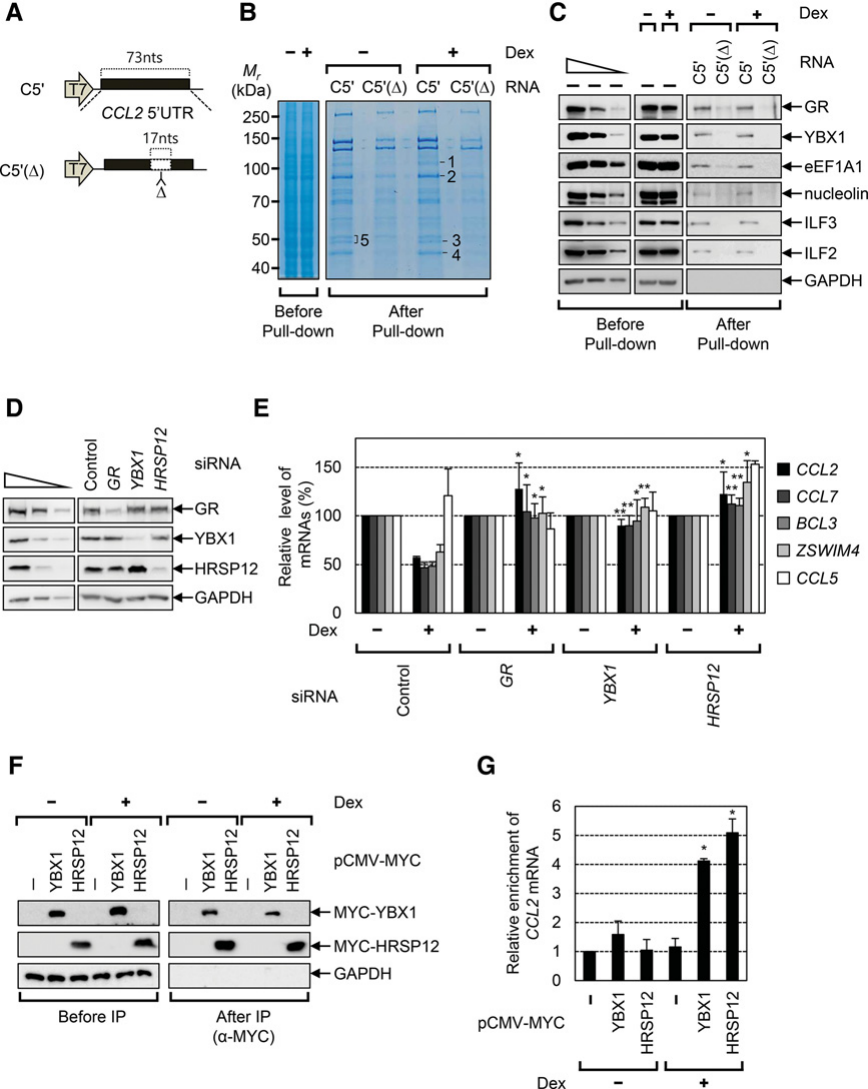
YBX1 and HRSP12 associate with mRNAs harboring a GR-binding site and are essential for efficient GMD. (*A*) A schematic diagram of RNA probes used in the RNA pull-down assay. (C5′) *CCL2* 5′ UTR; (Δ) the internal deletion of 17 nt in *CCL2* 5′ UTR corresponding to the GR-binding site. (*B*) Coomassie blue staining of cellular proteins copurified with biotinylated RNA probes. The extracts of the cells either treated or not treated with Dex for 3 h were mixed with in vitro transcribed biotinylated C5′ and C5′Δ RNA probes. Copurified cellular proteins were analyzed by SDS-PAGE gel and stained with Coomassie blue. The specific bands, which were subjected to LC-MS/MS, are depicted with numbers. (*C*) Western blot of cellular proteins copurified with biotinylated RNA probes. (*D*,*E*) The effect of down-regulation of YBX1 and HRSP12 on GMD. HeLa cells were depleted of endogenous YBX1, HRSP12, or, as a positive control, GR. The levels of endogenous GMD substrates were then quantitated by qRT–PCR. (*D*) Western blot demonstrating specific down-regulation by siRNA. (*E*) qRT–PCR of endogenous GMD substrates. *n* = 3. (*F*) Immunoprecipitations of MYC-YBX1 and MYC-HRSP12. (*G*) Dex-dependent enrichment of endogenous *CCL2* mRNAs in the immunoprecipitation of YBX1 and HRSP12. *n* = 2. (**) *P* < 0.01; (*) *P* < 0.05.

So far, several cellular factors involved in efficient GMD have been characterized: GR, UPF1, and PNRC2. To identify further GMD-specific factors, we performed an RNA pull-down assay using (1) extracts of the cells treated or not treated with Dex and (2) biotinylated *CCL2* 5′ UTR RNAs harboring either full-length (C5′ RNA) or a 17-nucleotide (nt) internal deletion lacking a GR-binding site [C5′(Δ) RNA] (Fig. 3A). The specific bands enriched in pull-down of biotinylated C5′ RNA, but not C5′(Δ) RNA, were eluted and subjected to liquid chromatography-tandem mass spectrometry (LC-MS/MS) (Fig. 3B). The newly identified proteins are in Supplemental Figure S3A. Specific interactions between the identified cellular proteins and C5′ RNA were further confirmed by Western blotting (Fig. 3C). All tested proteins (YBX1, eEF1A1, nucleolin, ILF2, and ILF3) were preferentially enriched in the pull-down of biotinylated C5′ RNA compared with the C5′(Δ) RNA. As expected, the specific enrichment of GR in the pull-down of biotinylated C5′ RNA was observed.

To determine the functional relevance of these factors in GMD, the cells were depleted of each factor using specific siRNAs. The levels of endogenous GMD target mRNAs encoding CCL2, CCL7, BCL3, or ZSWIM4 and the levels of *CCL5* mRNA, which served as a negative control, were then measured using qRT–PCR before and after Dex treatment. Whereas down-regulation of ILF2, ILF3, or eEF1A1 had no significant effect on GMD (Supplemental Fig. S3B–E), down-regulation of YBX1 almost completely blocked GMD (Fig. 3D,E), indicating that the RNA-binding protein YBX1 is a functional component in GMD.

Previously, YBX1 and the endoribonuclease HRSP12 have been characterized as cellular proteins associated with full-length *CCL2* mRNA (Dhawan et al. 2012). In the present study, we identified YBX1 as a *CCL2* 5′ UTR-interacting protein. Therefore, we asked whether HRSP12 also has the ability to trigger degradation of GMD substrates. The results showed that levels of all endogenous GMD substrates tested were increased when the cells were depleted of HRSP12 (Fig. 3D,E). All data indicate that YBX1 and HRSP12 are functionally involved in GMD.

### YBX1 and HRSP12 associate with *CCL2* mRNA in a ligand-dependent manner

Our previous data showed that, whereas GR binding to GMD substrates is independent of a ligand, the associations of PNRC2 and UPF1 with GMD substrates are promoted by a ligand. Therefore, we next asked whether YBX1 and HRSP12 associate with GMD substrates in a ligand-dependent manner. To this end, we performed immunoprecipitations using α-MYC antibody and the extracts of cells expressing either MYC-YBX1 or MYC-HRSP12 (Fig. 3F,G). After immunoprecipitations, the levels of coimmunoprecipitated endogenous *CCL2* mRNAs were analyzed using qRT–PCR. Specific immunoprecipitations were demonstrated by Western blotting (Fig. 3F). qRT–PCR results revealed that Dex treatment increased the amounts of the coimmunoprecipitated *CCL2* mRNAs in the immunoprecipitations of MYC-YBX1 and MYC-HRSP12 by fourfold and fivefold, respectively (Fig. 3G). All data indicate that a ligand promotes the association of YBX1 and HRSP12 with GMD substrates.

### YBX1 and HRSP12 are bona fide cellular factors for GMD

**Figure 4.**
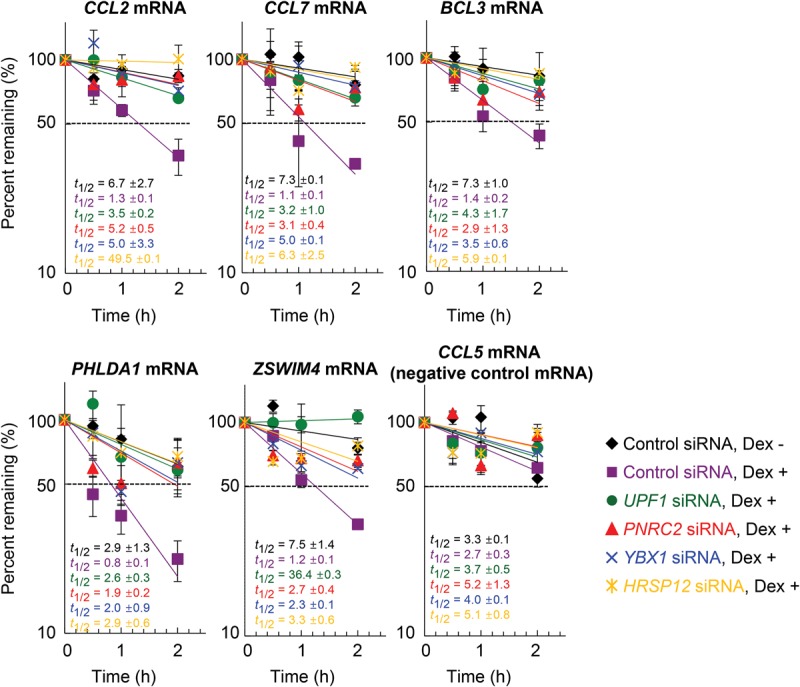
YBX1 and HRSP12 reduce the half-lives of endogenous GMD substrates. HeLa cells were depleted of endogenous UPF1, PNRC2, YBX1, or HRSP12 using specific siRNAs. To measure the half-lives of endogenous GMD substrates, the cells were treated with both 5,6-dichloro-1-β-D-ribofuranosylbenzimidazole (DRB; a potent transcription inhibitor) and Dex. Total cell RNAs were prepared at the indicated time points. The levels of endogenous GMD substrates, which were normalized to endogenous *GAPDH* mRNA, were plotted as a function of time after Dex treatment. The normalized levels of each GMD substrate at 0 h were arbitrarily set to 100%. *CCL5* mRNA, which lacks a GR-binding site, served as a negative control. The *Y*-axis represents the level of mRNA remaining (percentage) in logarithmic scale. The dots and bars represent the mean and standard deviation of two independent biological replicates.

We next tested whether YBX1 and HRSP12 are bona fide cellular factors for GMD. To this end, cells were specifically depleted of each GMD factor, which was confirmed by Western blotting (Supplemental Fig. S4), and the half-lives of endogenous GMD substrates were analyzed using qRT–PCR. Upon Dex treatment, the half-lives of all tested GMD substrates were drastically decreased. These decreases in half-life were significantly restored by down-regulation of UPF1, PNRC2, YBX1, or HRSP12 (Fig. 4), indicating that YBX1 and HRSP12 are bona fide cellular factors required for efficient GMD. Of note, the half-life of *CCL5* mRNA, which did not contain a GR-binding site and thus served as a negative control, was not significantly affected by Dex treatment and down-regulation of each GMD factor.

### Formation of a functionally active GMD complex requires the sequential recruitment of GMD factors

**Figure 5.**
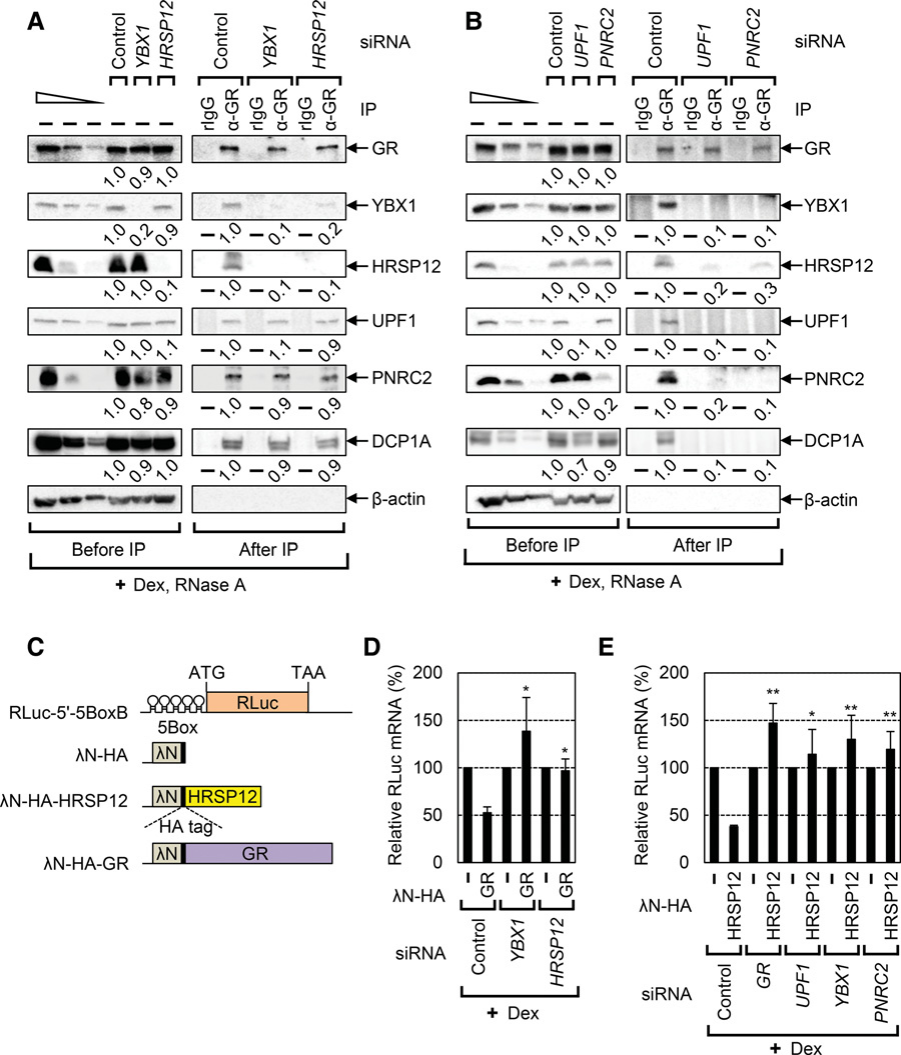
Sequential recruitment of GMD factors is involved in the formation of the active GMD complex. (*A*) Immunoprecipitation of endogenous GR using extracts of HEK293T cells depleted of either YBX1 or HRSP12. The cells were treated with Dex for 1 h before cell harvest. Total cell extracts were treated with RNase A to rule out RNA-mediated indirect interactions. Immunoprecipitations were performed using either α-GR antibody or, as a control, rabbit IgG (rIgG). The results are representative of two independently performed experiments. The levels of coimmunoprecipitated proteins were normalized to the level of immunoprecipitated GR. The normalized levels obtained from GR immunoprecipitation using undepleted cells were arbitrarily set to 1.0 (see also Supplemental Table S3 for details of quantitation). (*B*) Immunoprecipitation of endogenous GR using the extracts of the cells depleted of either UPF1 or PNRC2. This was performed as in *A* except that endogenous UPF1 or PNRC2 was down-regulated by siRNAs. *n* = 2. (*C*) A schematic diagram of the λN/5Box system. (*D*,*E*) GMD of the reporter mRNAs elicited by tethered GR (*D*) or HRSP12 (*E*). Cells were depleted of the indicated proteins using specific siRNAs, and then the tethering reporter and effector were transiently expressed. The cells were treated with Dex for 30 min before cell harvest. *n* = 3. (**) *P* < 0.01; (*) *P* < 0.05.

GMD occurs as two separable steps: substrate recognition by GR and rapid mRNA degradation induced by a ligand. Our previous report showed that GR is preloaded onto GMD substrates and that treatment with a ligand causes strong association of GR with PNRC2, UPF1, and DCP1A, eliciting rapid mRNA degradation (Cho et al. 2015). Therefore, we investigated how the newly identified GMD factors YBX1 and HRSP12 are recruited to target mRNAs and act in coordination with other GMD factors. To this end, we carried out several immunoprecipitation experiments using an antibody against endogenous GR (Fig. 5A,B).

First, newly identified GMD factors (YBX1 and HRSP12) as well as previously identified GMD factors (UPF1, PNRC2, and DCP1A) were enriched in the immunoprecipitation of endogenous GR in a process induced by Dex treatment (Supplemental Fig. S5A). Second, down-regulation of YBX1 or HRSP12 did not significantly affect the levels of coimmunoprecipitated UPF1, PNRC2, and DCP1A in the GR immunoprecipitation after the Dex and RNase A treatments (Fig. 5A). Intriguingly, down-regulation of YBX1 reduced the level of coimmunoprecipitated HRSP12 in GR immunoprecipitation by fivefold to 10-fold and vice versa. Third, down-regulation of UPF1 or PNRC2 caused a threefold to 10-fold reduction in the levels of all coimmunoprecipitated GMD factors tested (Fig. 5B). Consistent with a previous finding that UPF1 promotes an interaction between GR and PNRC2 (Cho et al. 2015), a fivefold to 10-fold smaller amount of coimmunoprecipitated PNRC2 was detected in the GR immunoprecipitation upon UPF1 down-regulation and vice versa. All of these results indicate that the formation of the GMD complex comprises at least two steps for the recruitment of GMD factors: (1) the mRNA-bound GR recruits UPF1 and PNRC2 in the presence of a ligand, and then (2) the GR–UPF1–PNRC2 complex recruits YBX1 and HRSP12 to complete the formation of the active GMD complex.

### The integrity of the GMD complex is necessary for GMD

It is known that artificially tethering GR to the 5′ UTR of a reporter mRNA triggers rapid mRNA degradation in the presence of a ligand (Cho et al. 2015). To determine a functional hierarchy of GMD factors, we employed the same tethering system coupled with specific down-regulation of GMD factors. In our tethering system, we used an RLuc reporter mRNA containing five tandem repeats of the bacteriophage BoxB sequence (5BoxB) in the 5′ UTR (Fig. 5C). GR and HRSP12 were N-terminally fused to λN-HA. Specific down-regulation by siRNA and the comparable expression of tethered proteins were confirmed by Western blotting (Supplemental Fig. S5B–E). Consistent with a previous report (Cho et al. 2015), artificially tethered GR reduced the level of the reporter mRNA by twofold in the presence of Dex. This reduction was completely restored upon down-regulation of YBX1 or HRSP12 (Fig. 5D).

Next, we tested the effect of tethered HRSP12 on the reporter mRNA. The result showed that artificially tethered HRSP12 reduced the levels of the reporter mRNA in the presence of Dex to the same extent as tethered GR. Intriguingly, the reduction by tethered HRSP12 was completely restored when the cells were depleted of GR, UPF1, YBX1, or PNRC2 (Fig. 5E). Considering that GMD complex formation is achieved by the sequential recruitment of GMD factors (Fig. 5A,B), these data indicate that all known GMD factors, including GR, UPF1, PNRC2, YBX1, and HRSP12, are necessary for a functionally active GMD complex. In other words, YBX1 and HRSP12, both of which are recruited to the complex in the last step, make the GMD complex functionally active.

### The residues for HRSP12 trimerization are important for the formation of a functionally active GMD complex

It has been shown that HRSP12, which is a member of the YER057c/YIL051c/YjgF family, forms a trimeric structure and has endoribonuclease activity (Morishita et al. 1999; Mistiniene et al. 2003, 2005). Although it is unknown whether the trimeric structure of HRSP12 is critical for its endoribonucleolytic activity, it is possible that the trimerization of HRSP12 is involved in maintaining the structural integrity of a functionally active GMD complex.

**Figure 6.**
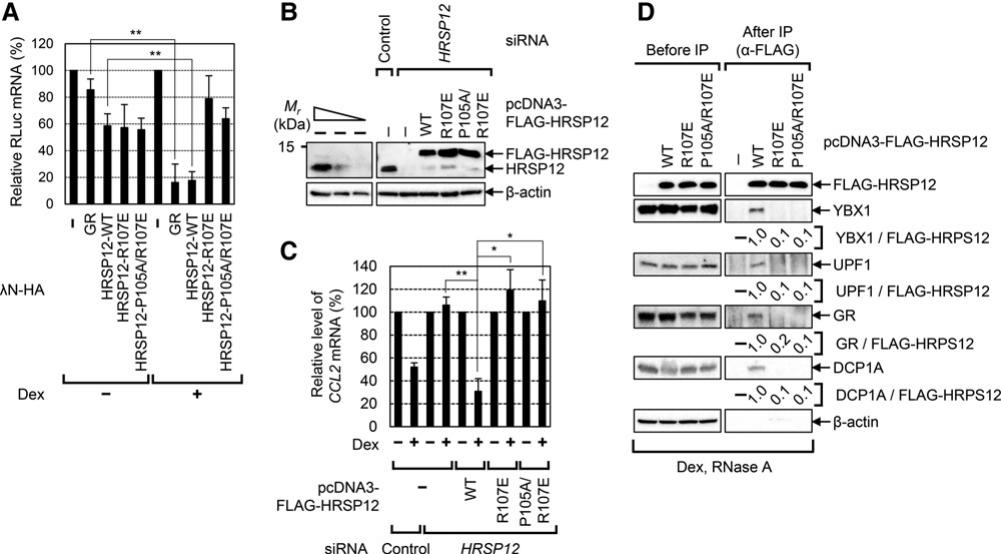
The residues for HRSP12 trimerization are essential for GMD. (*A*) GMD of the reporter mRNAs elicited by tethered HRSP12 either wild type or variants. Cells were transiently transfected with plasmids expressing tethering RLuc reporter mRNA and effector. The cells were treated with Dex for 30 min before cell harvest. The relative levels of the reporter RLuc mRNAs in the cells expressing λN-HA were arbitrarily set to 100%. *n* = 3. (**) *P* < 0.01. (*B*,*C*) Complementation experiments using *HRSP12* siRNA and Flag-HRSP12 either wild type or variants. (*B*) Specific down-regulation and comparable expression of HRSP12 were demonstrated by Western blotting. (*C*) GMD efficiency was measured by quantitating the level of endogenous *CCL2* mRNA. The levels of endogenous *CCL2* mRNA in the cells not treated with Dex for 1 h were arbitrarily set to 100%. *n* = 3. (**) *P* < 0.01; (*) *P* < 0.05. (*D*) Immunoprecipitations of Flag-HRSP12 either wild type or variants. Immunoprecipitations were performed using α-Flag antibody and the extracts of the cells transiently expressing Flag-HRSP12 either wild type or variants. The levels of coimmunoprecipitated proteins were normalized to the levels of immunoprecipitated Flag-HRSP12. The normalized levels obtained from the immunoprecipitation of Flag-HRSP12 wild type were arbitrarily set to 1.0. *n* = 2.

To test the above possibility, we constructed two variants: λN-HA-HRSP12-R107E and λN-HA-HRSP12-P105A/R107E. Previous data showed that these variants fail to form a trimeric structure (Mistiniene et al. 2005). The results of tethering experiments in which comparable expression of effectors was demonstrated by Western blotting (Supplemental Fig. S6) revealed that artificially tethered GR or HRSP12 wild type drastically reduced the levels of reporter mRNAs in a Dex-dependent manner (Fig. 6A). In contrast, although artificially tethered HRSP12 variants slightly reduced the levels of reporter mRNAs (as did tethered HRSP12 wild type) in the absence of Dex, Dex-dependent reduction of the reporter mRNAs was not observed when HRSP12 variants were tethered, indicating the importance of the residues for HRSP12 trimerization in efficient GMD.

To further clarify the role of the residues for HRSP12 trimerization in GMD, we carried out complementation experiments using (1) *HRSP12* siRNA targeting the 3′ UTR of *HRSP12* mRNA and (2) exogenously expressed Flag-tagged HRSP12 wild type or its variants. Comparable expression of endogenous HRSP12 and exogenously expressed Flag-HRSP12 was confirmed by Western blotting (Fig. 6B). Down-regulation of HRSP12 inhibited GMD of endogenous *CCL2* mRNA (Fig. 6C). Under the same conditions, when the Flag-HRSP12 wild type was exogenously expressed, the GMD efficiency of *CCL2* mRNA was completely restored. On the other hand, exogenous expression of Flag-HRSP12-R107E and Flag-HRSP12-P105A/R107E failed to restore the GMD efficiency. All data indicate that the residues for HRSP12 trimerization are essential for efficient GMD.

To investigate the underlying molecular mechanism of the way in which the residues for HRSP12 trimerization contribute to efficient GMD, we performed immunoprecipitations using either Flag-HRSP12 wild type or its variants (Fig. 6D). The immunoprecipitation results showed that endogenous YBX1, UPF1, GR, and DCP1A preferentially coimmunoprecipitated with Flag-HRSP12 wild type compared with Flag-HRSP12-R107E and Flag-HRSP12-P105A/R107E, indicating that the residues for HRSP12 trimerization are essential for the formation of a functionally active GMD complex.

### GMD targets diverse cellular transcripts, implicating a role in many cellular processes, including immune responses

**Figure 7.**
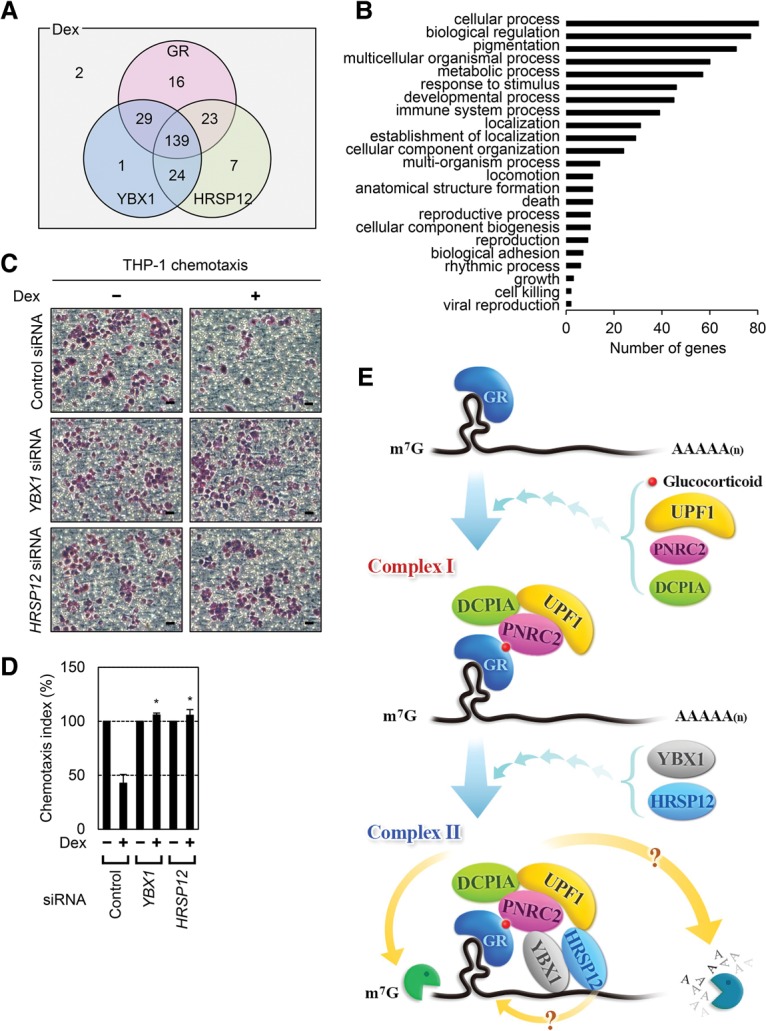
Genome-wide analysis identifies endogenous GMD substrates. (*A*) Venn diagrams representing the number of differentially expressed transcripts. HeLa cells depleted of GR, YBX1, or HRSP12 were either treated or not treated with Dex for 1 h. Total-cell RNAs were purified and subjected to mRNA-seq. Transcripts obtained from two independent biological replicates were analyzed and selected using the following criteria: at least twofold down-regulation upon Dex treatment and at least 1.5-fold up-regulation upon down-regulation of GR, YBX1, or HRSP12. (*B*) Gene ontology analyses using the WEGO software of 139 transcripts that were down-regulated by at least twofold upon Dex treatment and commonly up-regulated by at least 1.5-fold upon down-regulation of GR, YBX1, or HRSP12. (*C*,*D*) The effect of YBX1 and HRSP12 on chemotaxis of THP-1 cells. (*C*) The cells adherent to the membrane were stained. Bars, 25 µm. (*D*) The chemotaxis index was calculated by counting the stained cells. The chemotaxis index values obtained in the absence of Dex were arbitrarily set to 100%. *n* = 2. (*) *P* < 0.05. (*E*) Model illustrating the sequential recruitment of GMD factors. The details are described in the Discussion.

So far, a few GMD target mRNAs have been characterized. To identify additional GMD target substrates, we performed mRNA sequencing (mRNA-seq) using total RNAs purified from HeLa cells, which were depleted of each GMD factor and either treated or not treated with Dex. Specific down-regulation by siRNA was confirmed by Western blotting (Supplemental Fig. S7A). The genome-wide analysis of two independently performed transfections, mRNA isolation, and mRNA-seq showed that 241 transcripts (1% of the analyzed transcriptome) were down-regulated by at least twofold upon Dex treatment (Fig. 7A). Of these, 207, 193, and 193 transcripts were up-regulated by at least 1.5-fold upon down-regulation of GR, YBX1, and HRSP12, respectively. Notably, 139 out of 241 transcripts (57%) were up-regulated in all three cases (Fig. 7A; Supplemental Table S1). Among the commonly up-regulated transcripts was the previously identified GMD substrate *CCL2* mRNA.

Next, we confirmed mRNA-seq data by selecting interleukin 8 (*IL8*) mRNA, one of the mRNAs listed in the overlapped transcripts, and demonstrated that *IL8* mRNA is a bona fide GMD target mRNA. This was demonstrated by an increase in the level and half-life of *IL8* mRNA upon down-regulation of each GMD factor (Supplemental Fig. S7B,C) and by the selective enrichment of *IL8* mRNA in GR immunoprecipitation (Supplemental Fig. S7D,E).

We also performed gene ontology analysis of these overlapped transcripts. Among 139 transcripts, 119 (86%) of these genes are enriched in several distinct gene clusters, including cellular processes, biological regulation, and immune responses (Fig. 7B). These data indicate that GMD plays a role in regulating the stability of a variety of cellular transcripts.

### YBX1 and HRSP12 modulate the efficiency of chemotaxis of human monocytes

Our genome-wide analysis implied that GMD is involved in many diverse cellular functions (Fig. 7A,B). Indeed, our previous data showed that down-regulation of GMD factors UPF1, PNRC2, or GR promotes the chemotaxis of human acute monocytic leukemia cell lines (THP-1 cells) by up-regulating *CCL2* mRNA and CCL2 protein (Cho et al. 2015). These observations led us to investigate whether the newly identified GMD factors in this study, YBX1 and HRSP12, also play a role in chemotaxis of THP-1 cells by targeting *CCL2* mRNA (Fig. 7C,D). To this end, we monitored the efficiency of THP-1 cell migration using a chemotaxis microchamber, which was separated into two compartments by a membrane: (1) The upper compartment contained THP-1 cells, and (2) the lower compartment contained culture medium obtained from cells either depleted or not depleted of YBX1 and HRSP12 in the presence or absence of Dex. THP-1 cells, which express the CCL2 receptor, should respond to CCL2 protein in the lower compartment, migrate toward the lower compartment, and consequently adhere to the membrane.

The results showed that, when the lower compartment contained culture medium obtained from cells treated with Dex, the number of migrating cells (the number of cells adherent to the membrane; chemotaxis index) was decreased to 40% of the untreated control (Fig. 7C,D). Intriguingly, such a decrease was completely restored by down-regulation of YBX1 or HRSP12. Specific down-regulation and the CCL2 protein level in the culture medium were confirmed by Western blotting (Supplemental Fig. S8A) and enzyme-linked immunosorbent assay (ELISA) (Supplemental Fig. S8B), respectively. All data indicate that newly identified YBX1 and HRSP12 target the stability of *CCL2* mRNA, regulating chemotaxis of monocytes.

## Discussion

GMD is a recently defined new type of mRNA decay pathway, which is induced by a GR-specific ligand, GC (Cho et al. 2015). In this study, we identify new factors specific for GMD and characterize molecular details of the way in which GMD triggers target mRNA degradation. Based on our observations, we propose a model for the sequential recruitment of GMD factors to complete the formation of the active GMD complex (Fig. 7E). GMD can be divided into two steps: substrate recognition occurring in the absence of GC and rapid mRNA degradation occurring in the presence of GC. Since the binding efficiency of GR to a target mRNA is not affected by GC (Cho et al. 2015), GR would be preloaded onto a target mRNA in a sequence-dependent manner in the absence of GC. Next, the GR-bound mRNA would be subjected to rapid mRNA degradation in the presence of GC, which is preceded by sequential recruitment of GMD factors. First, GC binding to the GR preloaded onto mRNA promotes PNRC2 recruitment via direct interaction between the GR and PNRC2. The recruited PNRC2 provides a binding platform for UPF1 and DCP1A to form “GMD complex I,” which contains GC-bound GR, PNRC2, UPF1, and DCP1A. This complex is relatively stable, based on our immunoprecipitation results (Fig. 5A,B). However, with respect to GMD function, complex I is not fully active (Fig. 5C–E). Complex I would further recruit YBX1 and HRSP12 to form “GMD complex II.” This step may require the trimeric structure of HRSP12 to maintain structural integrity (Fig. 6). Such hierarchical recruitment of GMD factors leads to the formation of a functionally active GMD complex.

GMD complex II consists of complex I components, YBX1, and HRSP12. Among these, two factors, DCP1A and HRSP12, are known to be related to RNA-degrading ability: DCP1A promotes decapping activity of DCP2 (Coller and Parker 2004), and HRSP12 has an endoribonucleolytic ability (Morishita et al. 1999). Several previous studies and the present data support the important roles of DCP1A-mediated decapping and HRSP12-mediated endoribonucleolytic cleavage in GMD. Our previous complementation data showed that GMD inhibition by down-regulation of PNRC2 is restored by exogenously expressed PNRC2 wild type but not by PNRC2 mutants (which fail to interact with DCP1A), suggesting a critical role for DCP1A in GMD (Cho et al. 2015). In this study, the results of a tethering assay showed that down-regulation of HRSP12 blocked rapid mRNA degradation elicited by tethered GR (Fig. 5C–E) without affecting the formation of complex I (Fig. 5A,B). We also observed that down-regulation of HRSP12 completely blocks GMD efficiency, which is reversed by exogenously expressed HRSP12 (Figs. 3E, 6C), indicating an essential role for HRSP12 in GMD. Taking these data into account, it is therefore plausible that the decapping complex may act in coordination with the endoribonuclease HRSP12 to trigger rapid mRNA degradation during GMD. The functional connection between DCP1/2 and HRSP12 should be further investigated in the future.

Based on our observations, GMD is discriminated from NMD in several molecular aspects (Karam et al. 2013; Fatscher et al. 2015; Lykke-Andersen and Jensen 2015; Karousis et al. 2016). First, unlike NMD, GMD occurs independently of a translation event and EJC (Fig. 1). Second, whereas NMD requires the cooperative actions of UPF1-interacting effectors SMG5–7 and PNRC2, GMD preferentially uses PNRC2 (Fig. 2; Supplemental Fig. S2). Third, both GMD and NMD require helicase ability and phosphorylation of UPF1 (Fig. 2). However, for UPF1 phosphorylation, different PI3KKs—SMG1 and ATM—are preferentially involved in NMD and GMD, respectively (Fig. 2D; Supplemental Fig. S2C–E). ATM is a well-known PI3KK activated by DNA damage such as DNA double-strand breaks, which phosphorylates several target proteins associated with the DNA damage response (Marechal and Zou 2013). GC is one of the stress hormones that induces DNA damage (Flint et al. 2007; Lupien et al. 2007; Flint and Bovbjerg 2012; Reeder et al. 2015). The induced DNA damage could activate ATM kinase and phosphorylate UPF1 in the presence of GC, consequently enhancing GMD efficiency. Therefore, it is most likely that the preference of GMD for ATM may couple DNA damage responses to GMD target mRNA stability, contributing to the regulation of many physiologic events such as apoptosis (Schlossmacher et al. 2011).

Our genome-wide analysis indicates that GMD may play crucial roles in a variety of cellular processes (Fig. 7A,B), including the immune cell response (Fig. 7C,D). In particular, by way of half-life measurement and immunoprecipitation of GR, we demonstrated that *IL8* mRNA is a bona fide GMD target mRNA (Supplemental Fig. S7). It is known that IL8 is a multifunctional proinflammatory cytokine that functions in the chemotaxis, angiogenesis, and pathogenesis of bronchiolitis (Waugh and Wilson 2008). Considering that GC is a potent anti-inflammatory hormone, GC-induced GMD would counteract with IL8 functions. Therefore, further investigations of the relationship between GMD and its substrates will expand knowledge of the important roles of GMD in a wide range of physiological events.

## Materials and methods

### Plasmid construction

The details of plasmid construction are in the Supplemental Material.

### Cell culture and transfection

HeLa cells and HEK293T cells were cultured in DMEM (HyClone) containing 10% FBS (HyClone) and 1% penicillin/streptomycin (HyClone). The cells were transiently transfected with the plasmids using Lipofectamine2000 (Invitrogen). Two days after transfection, the cells were harvested, and total protein and RNA were purified as described previously (Kim et al. 2009). The details for down-regulation of endogenous protein using siRNA are in the Supplemental Material.

Where indicated, the cells were treated with 100 nM Dex (Sigma-Aldrich) for the specified time before cell harvesting. For the control of translational efficiency of IRE-containing reporter mRNAs, the cells were treated with either 50 µM hemin (Sigma-Aldrich) or 200 µM Df (Sigma-Aldrich) for 18 h before cell harvesting.

### qRT–PCR

qRT–PCR analyses were performed as described previously (Cho et al. 2012, 2015; Choe et al. 2014b). The oligonucleotides used in our study are in Supplemental Table S2.

### Measurement of mRNA half-lives

HeLa cells were transfected with 100 nM in vitro synthesized siRNA using oligofectamine. Three days later, the cells were treated with 100 µg/mL potent transcription inhibitor 5,6-dichloro-1-β-D-ribofuranosylbenzimidazole (DRB; Sigma-Aldrich) and 100 nM Dex for the specified time. The cells were then harvested, and total RNA was purified using TRIzol reagent (Life Technologies) at the time points indicated.

### Immunoprecipitations and RNA immunoprecipitations

HeLa cells and HEK293T cells were used in immunoprecipitations and RNA immunoprecipitations. The immunoprecipitations were performed as described previously (Cho et al. 2009; Kim et al. 2009). RNA immunoprecipitations were performed as described previously (Cho et al. 2015) except that Dynabeads Protein G (Life Technologies) were used without tRNA saturation. A primary antibody against GR (Abcam and BD Biosciences) was used for immunoprecipitations and RNA immunoprecipitations.

### Western blotting

Antibodies against the following proteins or peptides were used in this study: β-actin (Sigma-Aldrich, A5441), GAPDH (Ab Frontier, LF-PA0212), MYC (Calbiochem, OP10L), HA (Roche, 1867 431), UPF1 (a gift from Lynne E. Maquat, University of Rochester, Rochester, NY), PNRC2 (Cho et al. 2009), DCP1A (Cho et al. 2012), GR (Abcam, ab3579; and BD Biosciences, 611226), ABCE1 (Abcam, ab185548), SMG5 (Abcam, ab33033), SMG6 (Okada-Katsuhata et al. 2012), SMG7 (Bethyl Laboratories, A302-170A), YBX1 (Cell Signaling, D299), HRSP12 (Thermo Scientific, PA5-31352), eEF1A1 (Millipore, 05-235), nucleolin (Novus, NB600-241SS), and ILF2 (Bethyl Laboratories, A303-147A) and ILF3 (Bethyl Laboratories, A303-119). The intensities of each band after Western blotting were quantitated using Multigauge (Fuji Photo Film Co.) and then normalized to the intensities of either β-actin or the indicated protein. Quantitation results of Western blots in this study are summarized in Supplemental Table S3.

### CCL2 measurements and chemotaxis analysis

Concentrations of CCL2 proteins were measured according to the manufacturer's protocol (R&D System) using an ELISA kit. The chemotaxis assay was performed as described previously (Ko et al. 2004; Jang et al. 2007; Cho et al. 2015). In brief, chemotaxis assay was carried out using a 48-well chemotaxis microchamber (Neuroprobe). The two compartments in each well of the microchamber were separated by a polyvinylpyrrolidone-treated membrane (Neuroprobe) with 5-µm pores that was precoated with rat tail type I collagen overnight at 4°C. The lower and upper compartments were filled with cell supernatants and THP-1 cells, respectively. After incubation for 3 h at 37°C, the membranes were removed from the chamber. The nonadherent cells were removed by washing three times with phosphate-buffered saline (PBS). The adherent cells were fixed and stained in Diff-Quick staining solution (Baxter). The number of migrating (adherent) cells was counted under a microscope in six randomly selected visual fields in each well.

### Tethering assay

HeLa cells were transiently cotransfected with 0.1 µg of pRL-5′-5BoxB, 0.2 µg of a GR effector plasmid, 0.5 µg of HRSP12 effector plasmid, and 0.01 µg of pCI-F using Lipofectamine2000 (Invitrogen). Two days later, the cells were treated with 100 nM Dex for 30 min. The cells were then harvested, and total cell RNA and protein were purified using TRIzol reagent (Life Technologies).

### RNA pull-down assay

Biotin-labeled *CCL2* 5′ UTR (C5′) and *CCL2* 5′ UTR(Δ) (C5′Δ) were in vitro synthesized using XhoI-digested pSK-C5′ and pSK-C5′(Δ) as templates, T7 RNA polymerase (New England Biolabs), 2 mM NTPs, and 0.2 mM biotin-16-UTP (Roche). In vitro transcribed RNAs (15 µg) were incubated with tRNA-saturated streptavidin agarose resins (Thermo Scientific) in NET-2 buffer (50 mM Tris-HCl at pH 7.4, 150 mM NaCl, 1 mM PMSF, 2 mM benzamidine, 0.05% NP-40) containing 100 U of RNase inhibitor (Thermo Scientific) at 4°C.

For the preparation of cell extracts, HeLa cells were washed with ice-cold PBS and harvested by centrifugation at 3000*g* for 10 min at 4°C. Cell pellets were sonicated on ice with 2 × 30 bursts of 1 sec each (Branson Sonifier 250, output control 3, 30% duty cycle). After centrifugation at 13,000*g* for 10 min at 4°C, the supernatant was precleared by incubating with 50 µL of streptavidin resins for 1 h at 4°C. Precleared supernatant was mixed with in vitro transcribed RNA-bound streptavidin resin and incubated for 3 h at 4°C. The resins were washed five times with ice-cold NET-2 buffer. The resin-bound proteins were analyzed using 10% SDS-PAGE and were subjected to Coomassie blue staining or Western blotting.

### LC-MS/MS

Copurified proteins from the RNA pull-down assay were analyzed by SDS-PAGE gel and stained with Coomassie blue. In-gel digestion and LC-MS/MS were conducted by Proteomtech.

### mRNA-seq

HeLa cells transiently transfected with control siRNA or siRNA against GR, YBX1, or HRSP12 were treated or not treated with Dex for 1 h before cell harvest. Total-cell RNAs were purified using TRIzol and subjected to library constructions and mRNA-seq, which were provided by LAS. In brief, RNA integrity was measured with a bioanalyzer using an Agilent RNA 6000 Pico kit (Agilent). The isolated RNAs were further processed to prepare an mRNA-seq library using TruSeq stranded mRNA sample preparation kit (Illumina) according to the manufacturer's instructions. Sequencing of the library was carried out using an Illumina NextSeq500, and clusters of the cDNA libraries were generated on a flow cell and sequenced for 75-base-pair paired-end reads (2 × 75) with a NextSeq500 version 2 150-cycle kit (Illumina). The next-generation sequencing data have been deposited to the Sequence Read Archive of National Center for Biotechnology Information under accession number SRP078311.

### Statistical analysis

Two-tailed equal-sample variance Student's *t-*tests were used to calculate the *P*-values. Differences with *P* < 0.05 were considered statistically significant.

## Supplementary Material

Supplemental Material
