# Supplementary material for: Identification and molecular characterization of cellular factors required for glucocorticoid receptor-mediated mRNA decay
Source: Genes Dev. 2016 Sep 15;30(18):2093–105. doi: 10.1101/gad.286484.116 (PMC5066615; doi:10.1101/gad.286484.116)
Supplement: Supplemental Material [file supp_30_18_2093__index.html]

Supplemental Material 

# Identification and molecular characterization of cellular factors required for glucocorticoid receptor-mediated mRNA decay

## Supplemental Material

- Supplemental\_Figure\_S7.pdf
- Supplemental\_Figure\_S4.pdf
- Supplemental\_Figure\_S8.pdf
- Supplemental\_Figure\_S5.pdf
- Supplemental\_Material.docx
- Supplemental\_Figure\_S6.pdf
- Supplemental\_Table\_S1.xlsx
- Supplemental\_Figure\_S1.pdf
- Supplemental\_Table\_S2.xlsx
- Supplemental\_Figure\_S2.pdf
- Supplemental\_Table\_S3.xlsx
- Supplemental\_Figure\_S3.pdf
