# Supplementary figures and images for: Identification and molecular characterization of cellular factors required for glucocorticoid receptor-mediated mRNA decay
Source: Genes Dev. 2016 Sep 15;30(18):2093–105. doi: 10.1101/gad.286484.116 (PMC5066615; doi:10.1101/gad.286484.116)

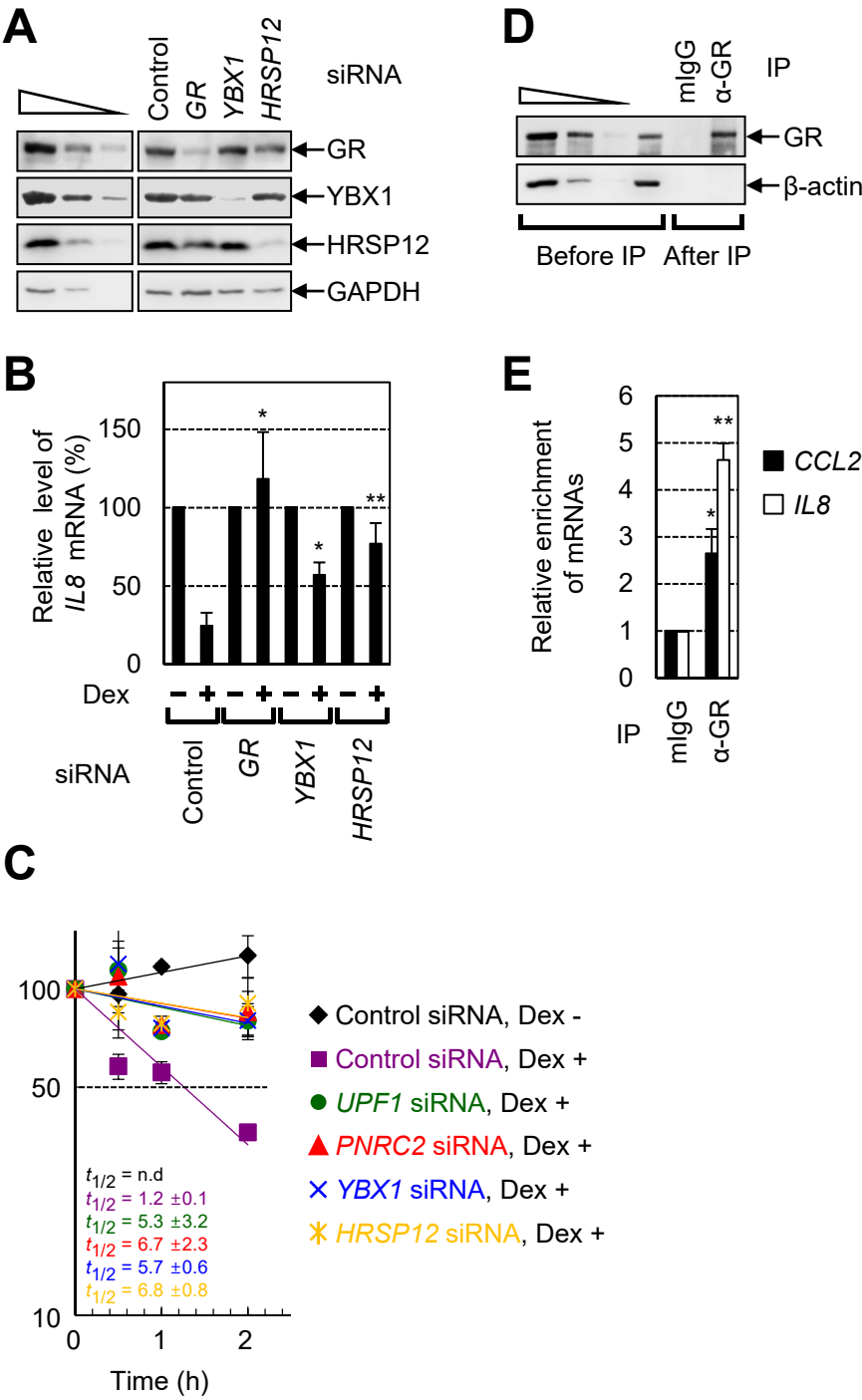

Supplement: Supplemental Material [file supp_30.18.2093_Supplemental_Figure_S7.pdf]

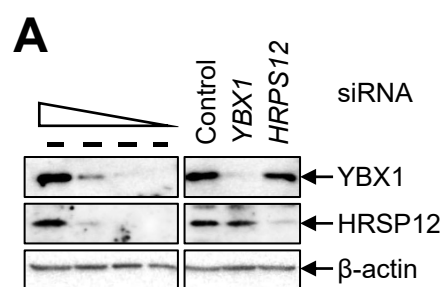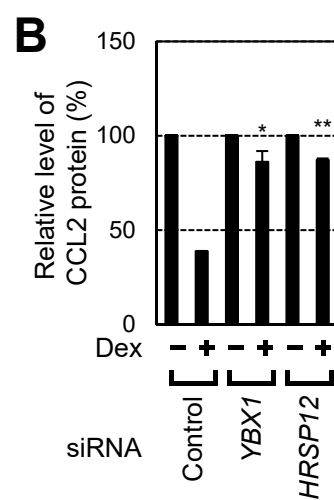

Supplement: Supplemental Material [file supp_30.18.2093_Supplemental_Figure_S8.pdf]

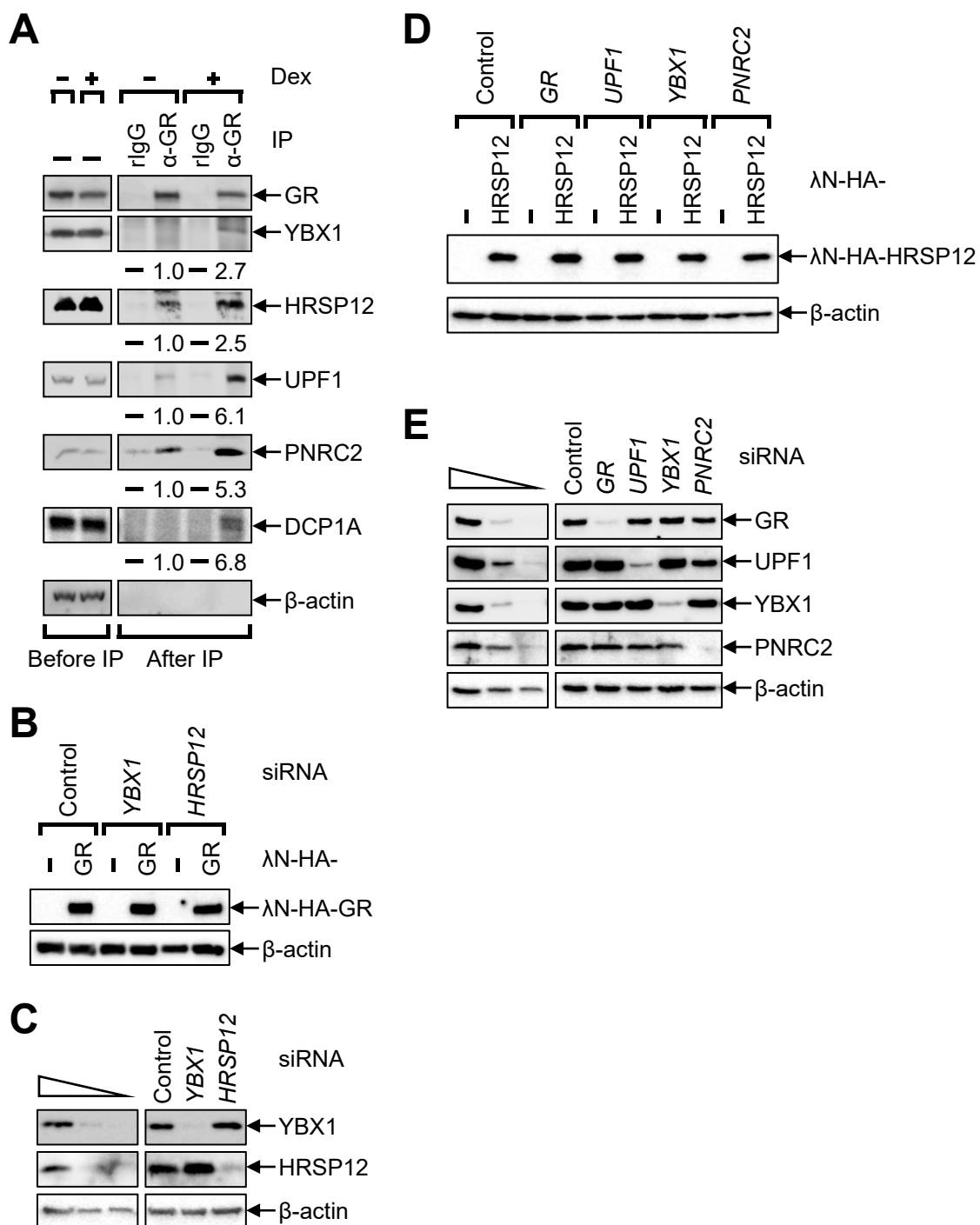

Supplement: Supplemental Material [file supp_30.18.2093_Supplemental_Figure_S5.pdf]

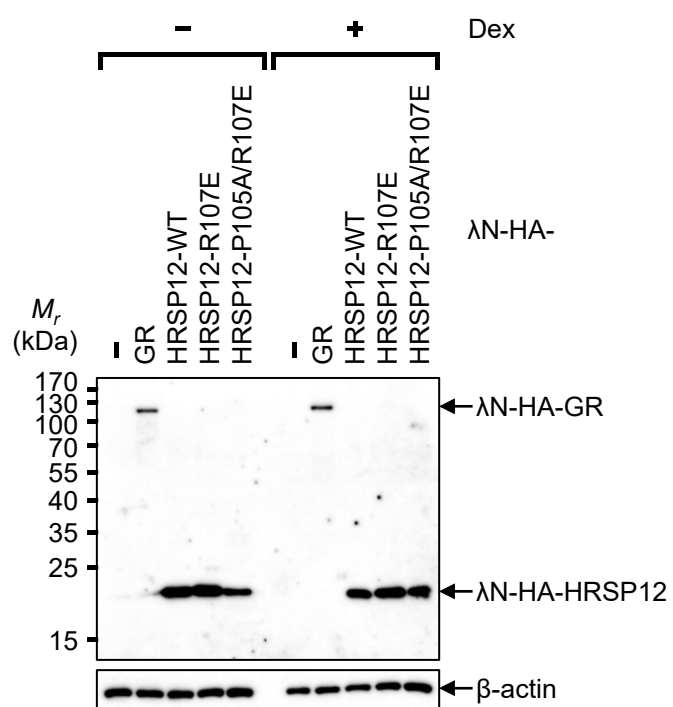

Supplement: Supplemental Material [file supp_30.18.2093_Supplemental_Figure_S6.pdf]

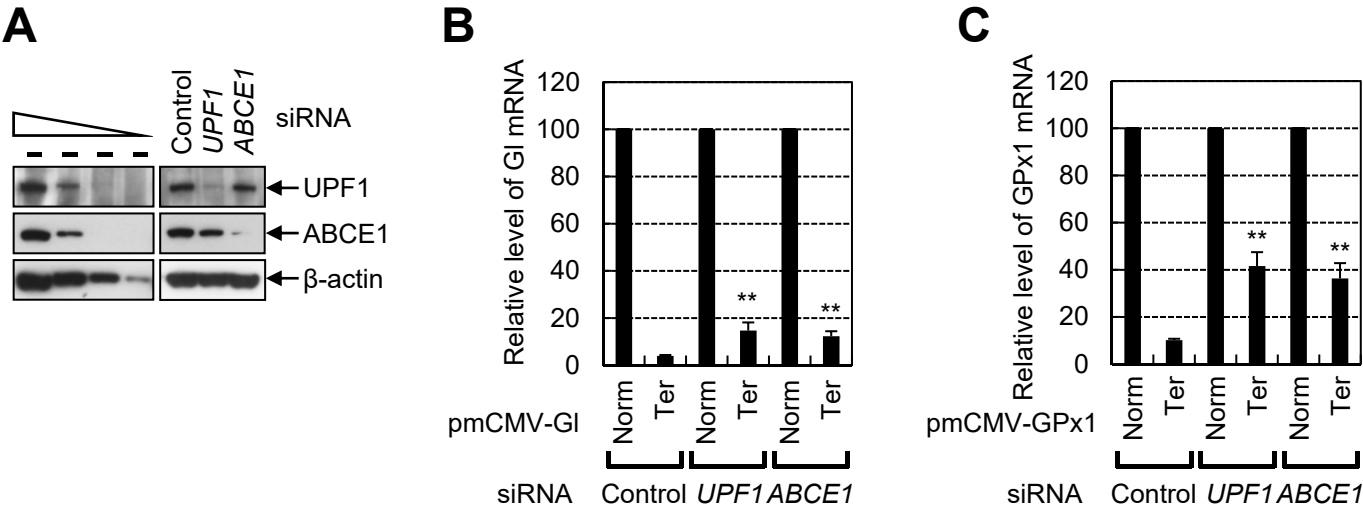

Supplement: Supplemental Material [file supp_30.18.2093_Supplemental_Figure_S1.pdf]

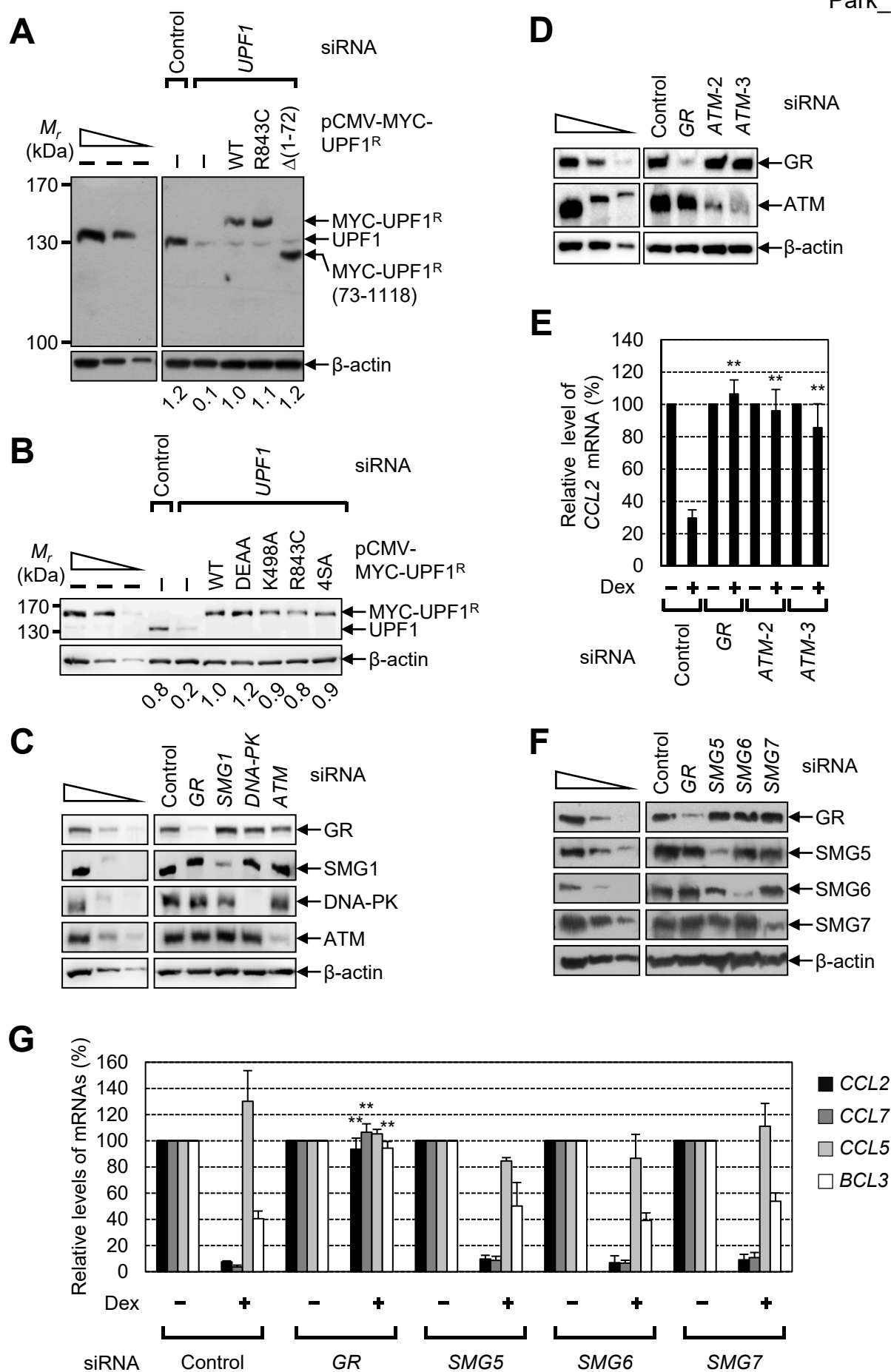

Supplement: Supplemental Material [file supp_30.18.2093_Supplemental_Figure_S2.pdf]
