# Supplementary material for: Identification and molecular characterization of cellular factors required for glucocorticoid receptor-mediated mRNA decay
Source: Genes Dev. 2016 Sep 15;30(18):2093–105. doi: 10.1101/gad.286484.116 (PMC5066615; doi:10.1101/gad.286484.116)
Supplement: Supplemental Material [file supp_30.18.2093_Supplemental_Material.docx]

**Supplemental Materials**

**Identification and molecular characterization of cellular factors required for glucocorticoid receptor-mediated mRNA decay**

**Ok Hyun Park^1,2^, Joori Park^1,2^, Mira Yu^1,2^, Hyoung-Tae An^2^, Jesang Ko^2^, and Yoon Ki Kim^1,2,*^**

^1^Creative Research Initiatives Center for Molecular Biology of Translation, Korea University, Seoul 02841, Republic of Korea

^2^Division of Life Sciences, Korea University, Seoul 02841, Republic of Korea

*Correspondence: [yk-kim@korea.ac.kr](mailto:yk-kim@korea.ac.kr); Tel: 82-2-3290-3410; FAX: 82-2-923-9923 (Y.K.K).

Running title: Molecular characterization of GMD

Key words: glucocorticoid receptor-mediated mRNA decay; YBX1; HRSP12; UPF1; PNRC2

**Supplemental Materials and Methods**

**Plasmid construction**

The following constructs have been reported previously: pCMV-MYC-UPF1^R^-WT and -R843C ([Isken et al. 2008](#_ENREF_6)), pIRE-Gl-Norm and -Ter ([Sato and Maquat 2009](#_ENREF_9)), pMS2-HA-PNRC2 ([Cho et al. 2009](#_ENREF_2)), pRβ-8bs-intron and pRβ-8bs-no intron ([Choe et al. 2014](#_ENREF_4)), and pRLuc-5′-5BoxB, pλN-HA, pλN-HA-GR, pC5′-RLuc, and pC5′(∆)-RLuc ([Cho et al. 2015](#_ENREF_3)).

To construct plasmid IRE-C5′-RLuc, the IRE sequences were amplified by polymerase chain reaction (PCR) using pIRE-Gl-Norm as template and specific oligonucleotides 5′-AACTGCAGACCCGGGTTTCCTGCTTCAACAGTG-3′ (sense) and 5′-AACTGCAGCCAGAGATTACTAGAGGTGTCTGTTTGAGGGTTCCGTCAAG-3′ (antisense), where the underlined nucleotides specify the PstI site. The PCR-amplified IRE structure was digested with PstI and ligated to a PstI-fragment of pC5′-RLuc.

To construct plasmids C5ʹ-RL-gGl and C5ʹ-RL-cGl, the BstBI/NotI fragment of either pRβ-8bs-intron or pRβ-8bs-no intron, which contained RLuc cDNA lacking a translation termination codon and either genomic sequence (gGl) or cDNA sequence (cGl) of β-globin gene sequence, respectively, was ligated to a BstBI/NotI fragment of pC5′-RLuc containing *CCL2* 5′UTR.

For the RNA pull-down assay, we constructed pSK-C5′ and pSK-C5′(∆), both of which contained a T7 promoter followed by the full-length and 17 nts-internal deletion of *CCL2* 5′UTR, respectively. A KpnI/XhoI fragment of pBluescript SK(-) (Stratagene) was ligated to a KpnI/XhoI fragment containing either the full-length or 17 nts-internal deletion of *CCL2* 5′UTR. The *CCL2* 5′ UTR fragments was amplified using pC5′-RLuc as a template and specific oligonucleotides: 5′-GGGGTACCGAGGAACCGAGAGGCTGAGACTAAC-3′ (sense) and 5′-CCGCTCGAGGCTGGAGGCGAGAGTGCGAGCTT-3′ (antisense), where the underlined nucleotides specify the KpnI and XhoI sites. The *CCL2* 5′UTR containing 17 nts-internal deletion was amplified using pC5′(∆)-RLuc as a template and specific oligonucleotides: 5′-GGGGTACCGAGGAACCGAGAGGCTGAGACTAACCCAGAAACATCCAAT-3′ (sense) and 5′-CCGCTCGAGGCTGGAGGCGAGAAGAATTGGATGTTTCTGGGTTAGTCTC-3′ (antisense), where the underlined nucleotides specify the KpnI and XhoI sites.

To construct plasmid pCMV-MYC-HRSP12, which expressed a human HRSP12 cDNA, an EcoRI/Acc65I fragment of pCMV-MYC (Clontech) was ligated to a PCR-amplified EcoRI/Acc65I fragment containing HRSP12 cDNA. HRSP12 cDNA was amplified by PCR using pCMV-SPORT6-HRSP12 (purchased from KRIBB) as a template and specific oligonucleotides: 5′-GGAATTCCGGATCCGATGTCGTCCTTGATCAGAAGGGTG-3′ (sense) and 5′-GGGGTACCTTATAGTGATGCCGTTGTCAGTGGTC-3′ (antisense), where the underlined nucleotides specify the EcoRI and Acc65I sites.

For artificial tethering of HRSP12 to 5′UTR of RLuc-5ʹ-5BoxB mRNA, pλN-HA-HRSP12-WT, which expressed λN-HA-fused human HRSP12 cDNA, was constructed by ligating the XbaI/NotI fragment of pλN-HA to a XbaI/NotI fragment of HRSP12 cDNA which was amplified by PCR using pCMV-MYC-HRSP12 as a template and specific oligonucleotides: 5′-GCTCTAGAGATCTCGTCCTTGATCAGAAG-3′ (sense) and 5′-TTGCGGCCGCTTATAGTGATGCCGTTGTCAG-3′ (antisense), where the underlined nucleotides specify the XbaI and NotI sites. Two HRSP12 variants, pλN-HA-HRSP12-R107E and pλN-HA-HRSP12-P105A/R107E were constructed using two-step PCR. All substitutions were confirmed by sequencing analysis.

To construct plasmid pCMV-MYC-YBX1, a fragment containing the full-length human YBX1 cDNA obtained from pCMV-SPORT6-YBX1 (purchased from KRIBB) was inserted into an XhoI/NotI sites of pCMV-MYC.

For complementation experiments, pCMV-MYC-UPF1^R^-73-1118, pCMV-MYC-UPF1^R^-DEAA, pCMV-MYC-UPF1^R^-K498A, and pCMV-MYC-UPF1^R^-4SA were generated, all of which expressed the siRNA-resistant (R) version of human UPF1 cDNA. All plasmids were constructed by either two-step PCRs or direct synthesis of DNA fragment containing amino acids S1073A, S1078A, S1096A and S1116A (Bioneer). All substitutions were confirmed by sequencing analysis.

For the complementation experiments using HRSP12, pcDNA3-FLAG-HRSP12-WT, -R107E, and P105A/R107E were constructed by ligating the BamHI/Acc65I fragment of pcDNA3-FLAG (a gift from Sung Key Jang at POSTECH, Korea) to a PCR-amplified BamHI/Acc65I fragment of HRSP12 cDNA, either WT or variant. All substitutions were confirmed by sequencing analysis.

**Downregulation of endogenous protein using siRNA**

To downregulate an endogenous protein using siRNA, HeLa cells or HEK293T cells were transfected with 100 nM of in vitro-synthesized siRNA (Gene Pharma) using Oligofectamine (Invitrogen). Three days after transfection, cells were harvested, and total protein and RNA were purified as described previously ([Kim et al. 2009](#_ENREF_7)).

The following siRNA sequences were used in this study: 5′-r(GAUGCAGUUCCGCUCCAUU)d(TT)-3′ for human UPF1 ([Kim et al. 2005](#_ENREF_8)), 5′-r(AGUUGGAAUUCUAGCUUAU)d(TT)-3′ for human PNRC2 ([Cho et al. 2009](#_ENREF_2)), 5′-r(GAGGAGAGUUGCAGAGAUU) d(TT)-3′ for human ABCE1, 5′-r(GUCAACCAUUGGAGGACAA) d(TT)-3′ for human eRF3 ([Hashimoto et al. 2012](#_ENREF_5)), 5′-r(CCUAUGUGCUGGAAGGAAU)d(TT)-3′ for human GR ([Cho et al. 2015](#_ENREF_3)), 5′-r(GCCAGAAAGAGGUGGGAAA)d(TT)-3′ for human SMG5 ([Cho et al. 2013](#_ENREF_1)), 5′-r(AAGCCAGUGAUACAGCGAAUU)d(TT)-3′ for human SMG6 ([Cho et al. 2013](#_ENREF_1)), 5′ r(GCAAGAAACAUCUGUGAUA)d(TT)-3′ for human SMG7 ([Cho et al. 2013](#_ENREF_1)), 5′-r(GGUCCUCCACGCAAUUACCAGCAAA)d(TT)-3′ for human YBX1, 5′-r(UGUAAUAGGGAGAGUUGAA)d(TT)-3′ for human HRSP12, 5′-r(GAGAGGAAGAAGAGGAGAA)d(TT)-3′ for human ILF2, 5′-r(CCAAGAAACCAAAGAAUGA)d(TT)-3′ for ILF3 and 5′-r(CCCAGGACACAGAGACUUU)d(TT)-3′ for human eEF1A1, 5′-r(AAGUGUAUGUGCGCCAAAGUA)d(TT)-3′ for human SMG1 ([Cho et al. 2015](#_ENREF_3)), 5′-r(AAGCGCCUGAUUCGAGAUCCU)d(TT)-3′ (*ATM*-1 siRNA)

5′-r(CGUGUCUUAAUGAGACUACAA)d(TT)-3′ (*ATM*-2 siRNA), 5′-r(UGAUGGUCUUAAGGAACAUCU)d(TT)-3′ (*ATM*-3 siRNA) for human ATM, 5′-r(CUUUAUGGUGGCCAUGGAG)d(TT)-3′ for human DNA-PKcs.

Supplemental Figure Legends

Supplemental Figure S1. Downregulation of ABCE1 inhibits NMD. HeLa cells depleted of UPF1 or ABCE1 were transfected with an NMD reporter plasmid and a control plasmid, phCMV-MUP. (*A*) Western blot demonstrating specific downregulation. (*B*) NMD of Gl mRNA. The relative levels of Gl mRNA were normalized to the levels of MUP mRNAs. The levels of normalized Gl Norm mRNA were set to 100%. (*C*) NMD of GPx1 mRNA. The columns and bars in each panel represent the mean and standard deviation of three independent biological replicates (n = 3). **, *P* < 0.01.

**Supplemental Figure S2.** Data supporting Fig. 2 and ATM-mediated UPF1 phosphorylation. (*A*,*B*) Western blot demonstrating specific downregulation of endogenous UPF1 and comparable expression of endogenous UPF1, exogenous UPF1^R^-WT, and its variants in Fig. 2B,C. (*C*) Western blots demonstrating specific downregulation in Fig. 2D. (*D*,*E*) GMD inhibition by downregulation of ATM using alternative siRNAs against ATM. (*D*) Specific downregulation of ATM was demonstrated by Western blotting. (*E*) The levels of endogenous *CCL2* mRNA were measured using qRT-PCR. n = 3; **, *P* < 0.01. (*F*,*G*) Effect of downregulation of SMG5, SMG6, or SMG7 on GMD. (*F*) Specific downregulation of SMG5, SMG6, and SMG7. (*G*) GMD efficiencies of endogenous GMD substrates. n = 3; **, *P* < 0.01.

**Supplemental Figure S3.** Identification of specific factors for GMD. (*A*) List of proteins identified by RNA pull-down followed by LC-MS/MS. (*B*-*E*) HeLa cells were depleted of the indicated protein using siRNA, and levels of endogenous GMD substrate, *CCL2* mRNA, were measured using qRT-PCR (*C*,*E*). Specific downregulation by siRNA transfection were confirmed by Western blotting (*B*,*D*). n = 3; **, *P* < 0.01; *, *P* < 0.05.

**Supplemental Figure S4.** Specific downregulation by siRNAs in half-life experiments. The protein samples in Fig. 4 were subjected to Western blotting to demonstrate specific downregulation.

**Supplemental Figure S5.** Enhanced formation of GMD complex in the presence of Dex and confirmation of expression levels and downregulation efficiencies. (*A*) IP of endogenous GR using the extracts of the cells either treated or not treated with Dex for 1 hr. n = 2. (*B*-*E*) The protein samples in Fig. 5D,E were subjected to Western blotting to demonstrate proper expression of tethered proteins (*B*,*D*) and specific downregulation by the indicated siRNAs (*C*,*E*).

**Supplemental Figure S6.** Confirmation of expression levels of tethered proteins. The protein samples in Fig. 6A were subjected to Western blotting to demonstrate proper expression of tethered proteins.

**Supplemental Figure S7.** *IL8* mRNA is a bona fide GMD substrate. (*A*,*B*) Effect of YBX1 and HRSP12 on the level of endogenous *IL8* mRNA. The experiments were performed as in Fig. 3D,E. n = 3; **, *P* < 0.01; *, *P* < 0.05. (*C*) Effect of YBX1 and HRSP12 on the half-life of *IL8* mRNA. The experiments were performed as in Fig. 4. n = 2. (*D*,*E*) RNA IP using α-GR antibody. IPs were performed using α-GR antibody and the extracts of the HeLa cells. The levels of immunoprecipitated GR and co-immunoprecipitated mRNAs were analyzed by Western blotting (*D*) and qRT-PCR (*E*), respectively. n = 2; **, *P* < 0.01; *, *P* < 0.05.

**Supplemental Figure S8.** Demonstration of specific downregulation by siRNA and the measurement of CCL2 protein. (*A*) Western blotting of YBX1 and HRSP12, demonstrating specific downregulation shown in Fig. 7C,D. (*B*) ELISA to monitor the levels of CCL2 protein in the supernatants of the HeLa cells depleted of either YBX1 or HRSP12 shown in Fig. 7C,D. n = 2.

**Supplemental Table S1.** List of 139 transcripts that were downregulated by at least 2-fold upon Dex treatment, and commonly upregulated by at least 1.5-fold upon downregulation of GR, YBX1, or HRSP12.

**Supplemental Table S2.** Oligonucleotides used for qRT-PCR in this study.

**Supplemental Table S3.** Quantitation results of Western blots in this study. The intensities of each band after Western blotting were quantitated using Multigauge. The mean and standard deviation of relative intensities are depicted in the dataset.

**Supplemental References**

Cho H, Han S, Choe J, Park SG, Choi SS, Kim YK. 2013. SMG5-PNRC2 is functionally dominant compared with SMG5-SMG7 in mammalian nonsense-mediated mRNA decay. *Nucleic Acids Res* **41**: 1319-1328.

Cho H, Kim KM, Kim YK. 2009. Human proline-rich nuclear receptor coregulatory protein 2 mediates an interaction between mRNA surveillance machinery and decapping complex. *Mol Cell* **33**: 75-86.

Cho H, Park OH, Park J, Ryu I, Kim J, Ko J, Kim YK. 2015. Glucocorticoid receptor interacts with PNRC2 in a ligand-dependent manner to recruit UPF1 for rapid mRNA degradation. *Proceedings of the National Academy of Sciences of the United States of America* **112**: E1540-1549.

Choe J, Ryu I, Park OH, Park J, Cho H, Yoo JS, Chi SW, Kim MK, Song HK, Kim YK. 2014. eIF4AIII enhances translation of nuclear cap-binding complex-bound mRNAs by promoting disruption of secondary structures in 5'UTR. *Proceedings of the National Academy of Sciences of the United States of America* **111**: E4577-4586.

Hashimoto Y, Hosoda N, Datta P, Alnemri ES, Hoshino S. 2012. Translation termination factor eRF3 is targeted for caspase-mediated proteolytic cleavage and degradation during DNA damage-induced apoptosis. *Apoptosis : an international journal on programmed cell death* **17**: 1287-1299.

Isken O, Kim YK, Hosoda N, Mayeur GL, Hershey JW, Maquat LE. 2008. Upf1 phosphorylation triggers translational repression during nonsense-mediated mRNA decay. *Cell* **133**: 314-327.

Kim KM, Cho H, Choi K, Kim J, Kim BW, Ko YG, Jang SK, Kim YK. 2009. A new MIF4G domain-containing protein, CTIF, directs nuclear cap-binding protein CBP80/20-dependent translation. *Genes Dev* **23**: 2033-2045.

Kim YK, Furic L, Desgroseillers L, Maquat LE. 2005. Mammalian Staufen1 recruits Upf1 to specific mRNA 3'UTRs so as to elicit mRNA decay. *Cell* **120**: 195-208.

Sato H, Maquat LE. 2009. Remodeling of the pioneer translation initiation complex involves translation and the karyopherin importin beta. *Genes Dev* **23**: 2537-2550.
