# Supplementary material for: Identification and molecular characterization of cellular factors required for glucocorticoid receptor-mediated mRNA decay
Source: Genes Dev. 2016 Sep 15;30(18):2093–105. doi: 10.1101/gad.286484.116 (PMC5066615; doi:10.1101/gad.286484.116)

A

Band #	Protein name	Accession No	Mass (kDa)	Sequence coverage (%)
1	tRNA (cytosine(34)-C(5))-methyltransferase isoform 1	gi|39995082	86	9
1	Pyruvate carboxylase	gi|189306	129	3
1	Nucleolin	gi|632808	76	3
1	DEAH (Asp-Glu-Ala-His) Box Helicase 9	gi|8129349	48	6
1	T cell receptor alpha chain V-J-region, partial	gi|902377536	71	12
2	Interleukin enhancer-binding factor 3	gi|1770458	90	16
3	Y-box-binding protein 1	gi|181486	39	20
4	Interleukin enhancer-binding factor 2	gi|532313	45	7
5	Y-box-binding protein 3	gi|181486	39	4
5	SS-B/La protein	gi|338497	72	15
5	Eukaryotic translation elongation factor 1 alpha	gi|181965	24	4

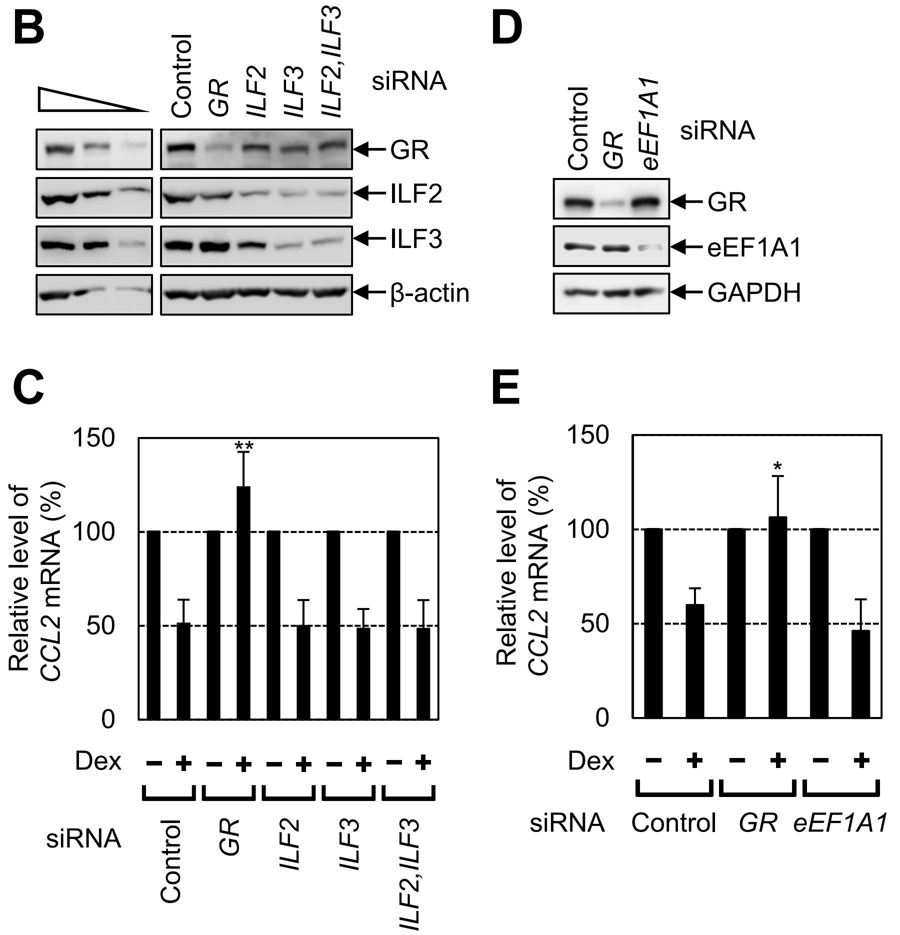

Supplement: Supplemental Material [file supp_30.18.2093_Supplemental_Figure_S3.pdf]
