# Supplementary material for: Identification and molecular characterization of cellular factors required for glucocorticoid receptor-mediated mRNA decay
Source: Genes Dev. 2016 Sep 15;30(18):2093–105. doi: 10.1101/gad.286484.116 (PMC5066615; doi:10.1101/gad.286484.116)
Supplement: Supplemental Material [file supp_30.18.2093_Supplemental_Figure_S4.pdf]

Park_Fig. S4

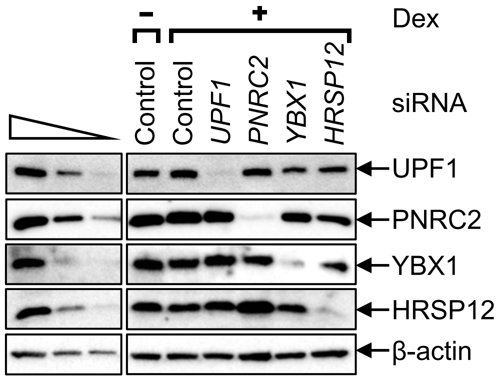

-
+
Dex
Control
Control
UPF1
PNRC2
YBX1
HRSP12
siRNA
UPF1
PNRC2
YBX1
HRSP12
β-actin
